# Cortical patterning of abnormal morphometric similarity in psychosis is associated with brain expression of schizophrenia-related genes

**DOI:** 10.1073/pnas.1820754116

**Published:** 2019-04-19

**Authors:** Sarah E. Morgan, Jakob Seidlitz, Kirstie J. Whitaker, Rafael Romero-Garcia, Nicholas E. Clifton, Cristina Scarpazza, Therese van Amelsvoort, Machteld Marcelis, Jim van Os, Gary Donohoe, David Mothersill, Aiden Corvin, Andrew Pocklington, Armin Raznahan, Philip McGuire, Petra E. Vértes, Edward T. Bullmore

**Affiliations:** ^a^Department of Psychiatry, University of Cambridge, Cambridge CB2 0SZ, United Kingdom;; ^b^Developmental Neurogenomics Unit, National Institute of Mental Health, Bethesda, MD 20892;; ^c^The Alan Turing Institute, London NW1 2DB, United Kingdom;; ^d^Neuroscience and Mental Health Research Institute, Cardiff University, Cardiff CF24 4HQ, United Kingdom;; ^e^Medical Research Council Centre for Neuropsychiatric Genetics and Genomics, Institute of Psychological Medicine and Clinical Neurosciences, Cardiff University, Cardiff CF24 4HQ, United Kingdom;; ^f^Department of Psychosis Studies, Institute of Psychiatry, Psychology and Neuroscience, King’s College London, London SE5 8AF, United Kingdom;; ^g^Department of General Psychology, University of Padova, 35131 Padova, Italy;; ^h^Department of Psychiatry and Neuropsychology, Maastricht University, 616 6200, Maastricht, The Netherlands;; ^i^Department of Psychiatry, University Medical Center Utrecht Brain Center, 3584 CG, Utrecht, The Netherlands;; ^j^School of Psychology, National University of Ireland Galway, Galway H91 TK33, Ireland;; ^k^Department of Psychiatry, Trinity College Dublin, Dublin 8, D08 W9RT, Ireland;; ^l^School of Mathematical Sciences, Queen Mary University of London, London E1 4NS, United Kingdom;; ^m^ImmunoPsychiatry, GlaxoSmithKline R&D, Stevenage SG1 2NY, United Kingdom

**Keywords:** dysconnectivity, psychosis, network neuroscience, morphometric similarity, Allen Human Brain Atlas

## Abstract

Schizophrenia has been conceived as a disorder of brain connectivity, but it is unclear how this network phenotype is related to the underlying genetics. We used morphometric similarity analysis of MRI data as a marker of interareal cortical connectivity in three prior case–control studies of psychosis: in total, *n* = 185 cases and *n* = 227 controls. Psychosis was associated with globally reduced morphometric similarity in all three studies. There was also a replicable pattern of case–control differences in regional morphometric similarity, which was significantly reduced in patients in frontal and temporal cortical areas but increased in parietal cortex. Using prior brain-wide gene expression data, we found that the cortical map of case–control differences in morphometric similarity was spatially correlated with cortical expression of a weighted combination of genes enriched for neurobiologically relevant ontology terms and pathways. In addition, genes that were normally overexpressed in cortical areas with reduced morphometric similarity were significantly up-regulated in three prior post mortem studies of schizophrenia. We propose that this combined analysis of neuroimaging and transcriptional data provides insight into how previously implicated genes and proteins as well as a number of unreported genes in their topological vicinity on the protein interaction network may drive structural brain network changes mediating the genetic risk of schizophrenia.

## Significance

Despite significant research, the biological mechanisms underlying schizophrenia are still unclear. We shed light on structural brain differences in psychosis using an approach called morphometric similarity mapping, which quantifies the structural similarity between brain regions. Morphometric similarity was globally reduced in psychosis patients in three independent datasets, implying that patients’ brain regions were more differentiated from each other and less interconnected. Similarity was especially decreased in frontal and temporal regions. This anatomical pattern was correlated with expression of genes enriched for nervous system development and synaptic signaling and genes previously associated with schizophrenia and antipsychotic treatments. Therefore, we begin to see how combining genomics and imaging can give a more integrative understanding of schizophrenia, which might inform future treatments.

Psychotic disorders have a lifetime prevalence of 1–3% and can be extremely debilitating. However, despite significant efforts, the brain architectural changes and biological mechanisms causing psychotic disorders are not yet well understood, and there has been correspondingly limited progress in the development of new therapeutics.

MRI studies of schizophrenia have robustly demonstrated local structural differences in multiple cortical areas, subcortical nuclei, and white matter tracts (1). The most parsimonious explanation of this distributed, multicentric pattern of structural change is that it reflects disruption or dysconnectivity of large-scale brain networks comprising anatomically connected brain areas. However, testing this dysconnectivity hypothesis of psychotic disorder has been constrained by the fundamental challenges in measuring anatomical connectivity and brain anatomical networks in humans. The principal imaging methods available for this purpose are tractographic analysis of diffusion weighted imaging (DWI) and structural covariance analysis of conventional MRI. DWI-based tractography generally underestimates the strength of long-distance anatomical connections: for example, between bilateral homologous areas of cortex. Structural covariance analysis is not applicable to single-subject analysis, and its biological interpretation is controversial (2).

We recently proposed a technique known as “morphometric similarity mapping” (3), which quantifies the similarity between cortical areas in terms of multiple MRI parameters measured at each area and can be used to construct whole-brain anatomical networks for individual subjects. In keeping with histological results indicating that cytoarchitectonically similar areas of cortex are more likely to be anatomically connected (4), morphometric similarity in the macaque cortex was correlated with tract-tracing measurements of axonal connectivity. Compared with both tractographic DWI-based networks and structural covariance networks, morphometric similarity networks included a greater proportion of connections between human cortical areas of the same cytoarchitectonic class. Individual differences in regional mean morphometric similarity or “hubness” of cortical nodes in morphometric similarity networks accounted for about 40% of the individual differences in intelligence quotient (IQ) in a sample of 300 healthy young people. These results suggest that morphometric similarity mapping could provide a useful tool to analyze psychologically relevant biological differences in brain structure.

Here, we used morphometric similarity mapping to test the dysconnectivity hypothesis of psychosis in three independent case–control MRI datasets: the Maastricht Genetic Risk and Outcome of Psychosis (GROUP) study (83 cases and 68 controls) and the Dublin study (33 cases and 82 controls), both made available as legacy datasets for the PSYSCAN project (5), as well as the publicly available Cobre dataset (69 cases and 77 controls) (*Materials and Methods*). We mapped case–control morphometric similarity differences at global and nodal levels of resolution individually in each dataset to assess replicability, and we tested for significant differences in network organization that were consistent across studies. We used partial least squares (PLS) regression to test the hypothesis that this MRI network phenotype of psychosis was correlated with anatomically patterned gene expression using data from the Allen Human Brain Atlas (AHBA). This analytical approach to combine imaging and genomic data has been methodologically established (6, 7) and applied in the context of neuropsychiatric disorders (8, 9). We used it to test the pathogenic hypothesis that the genes most strongly associated with case–control differences in morphometric similarity were enriched: (*i*) for genes that have been ontologically linked to relevant neurobiological processes and (*ii*) for genes that are abnormally expressed in post mortem studies of schizophrenia.

## Results

### Samples.

Sociodemographic and clinical data available on the sample are in *SI Appendix*, Table S1. There was considerable heterogeneity in clinical measures between studies (e.g., the Maastricht patients had relatively low mean scores on psychotic symptom scales).

### Case–Control Differences in Global Morphometric Similarity.

**Fig. 1.**
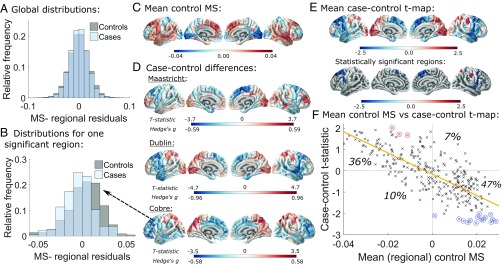
Case–control differences in regional morphometric similarity. (*A*) Case and control distributions of regional morphometric similarity strength, i.e., the average similarity of each region to all other regions, pooling data from all three primary studies. (*B*) Distributions of morphometric similarity strength for a region with significantly reduced morphometric similarity in cases, namely left hemisphere caudal middle frontal part 1. (*C*) Regional morphometric similarity averaged over controls from all three datasets. (*D*) t statistics and Hedge’s g effect sizes for the case–control differences in regional morphometric similarity in each dataset. (*E*) t statistics for regional case–control differences averaged across datasets in all regions and in the 18 cortical areas where the difference was statistically significant across datasets (FDR = 0.05). (*F*) Scatterplot of mean control regional morphometric similarity (x axis) vs. case–control t statistic (y axis). Control morphometric similarity (from *C*) is strongly negatively correlated with case–control morphometric similarity differences (from *D*; Pearson’s r = −0.76, P<0.001). Most cortical regions have positive morphometric similarity in controls, which decreases in cases (47% of regions), or negative morphometric similarity in controls, which increases in cases (36% of regions). Statistically significant regions are circled in red or blue according to whether their mean t statistic increases or decreases, respectively, in patients. MS, morphometric similarity.

Globally, morphometric similarity was reduced in cases compared with controls in all three datasets (*SI Appendix*, Fig. S2). Regional morphometric similarity had an approximately normal distribution over all 308 regions (after regressing age, sex, and age × sex) and, in all three datasets, there was a significant case–control difference in this distribution (P<0.001, Kolmogorov–Smirnoff test). Modal values of regional morphometric similarity were more frequent and extreme values were less frequent in cases compared with controls (Fig. 1*A* and *SI Appendix*, Fig. S2).

### Case–Control Differences in Regional Morphometric Similarity.

The cortical map of regional morphometric similarity in Fig. 1*C* summarizes the anatomical distribution of areas of positive and negative similarity on average over controls from all three datasets. The results are similar to those reported in an independent sample (3), with high positive morphometric similarity in frontal and temporal cortical areas and high negative morphometric similarity in occipital, somatosensory, and motor cortices. This confirms the replicability of this pattern of regional morphometric similarity in healthy individuals and is consistent with prior knowledge that primary cortex is more histologically differentiated than association cortex.

We mapped the t statistics and corresponding Hedge’s g effect sizes for the case–control differences in regional morphometric similarity at each cortical area (Fig. 1*D*). A positive t statistic means that morphometric similarity increased in patients, whereas a negative t statistic means that morphometric similarity decreased. We found somewhat similar patterns of case–control difference across all three datasets, with increased regional morphometric similarity in occipital and parietal areas in patients and decreased regional morphometric similarity in frontal and temporal cortices. The case–control t map for the Dublin study was significantly correlated with both the Maastricht and the Cobre t maps (r=0.42, P<0.001 and r=0.47, P<0.001, respectively), although the Maastricht and Cobre t maps were not significantly correlated (r=0.058, P=0.31) (*SI Appendix*, Fig. S4). However, a large number of patients in the Maastricht dataset had very low symptom scores [below the threshold for “borderline mentally ill” (10)]. If those nonpsychotic patients were excluded from the analysis, the Maastricht case–control t map was correlated significantly with the Cobre map (r = 0.22, P<0.001) (*SI Appendix*, section S6.2).

Combining the P values for case–control differences across all three datasets, we identified 18 cortical regions where morphometric similarity was robustly and significantly different between groups (Fig. 1*E*). Morphometric similarity decreased in patients in 15 regions located in the superior frontal, caudal middle frontal, precentral, pars triangularis, and superior temporal areas and increased in 3 regions located in superior parietal and postcentral areas (*SI Appendix*, Table S2).

To contextualize the regional morphometric similarity case–control differences, we referred them to two prior classifications of cortical areas: the von Economo atlas of cortex classified by cytoarchitectonic criteria (6) and the Yeo atlas of cortex classified according to resting-state networks derived from fMRI (11, 12). Morphometric similarity was significantly reduced in von Economo class 2 (association cortex) and in the ventral attention, frontoparietal, and default mode fMRI networks (all $P_{FDR}<0.05$) (*SI Appendix*, Tables S12 and S13).

There was a strong negative correlation between regional morphometric similarity in the control subjects and the case–control differences in regional morphometric similarity (both averaged over all three datasets; $P_{perm}=0.002$) (Fig. 1*F*). Hence, areas with the highest positive morphometric similarity in controls tended to show the greatest decrease of morphometric similarity in patients, and conversely, areas with the highest negative morphometric similarity in healthy controls had the greatest increase of morphometric similarity in psychosis. This result is analogous to the observation that highly connected “hub” regions are the most likely to show reduced connectivity in disease in fMRI and diffusion tensor imaging (DTI) brain networks (13).

We tested for correlations between mean morphometric similarity and a range of clinical measures, including symptom scores, antipsychotic medication use, and cannabis use (*SI Appendix*, section S6.3). The only significant associations after false discovery rate (FDR) correction were with cannabis use, which was positively correlated with mean global morphometric similarity in the Maastricht study ($P_{FDR}=5\times 10^{-4}$) as well as with mean morphometric similarity averaged across the 15 regions with significantly decreased morphometric similarity in Fig. 1*E* ($P_{FDR}$ = 0.0017).

### Gene Expression Related to Morphometric Similarity.

**Fig. 2.**
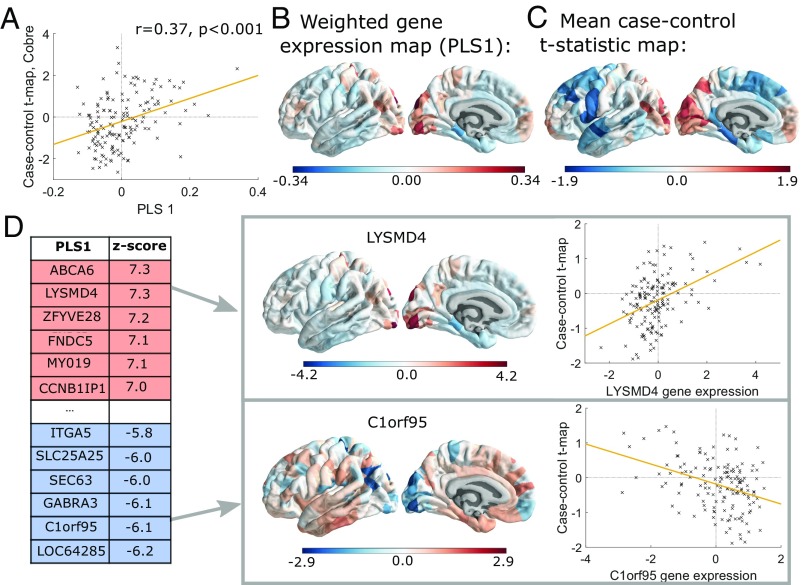
Gene expression profiles related to case–control differences in morphometric similarity. (*A*) Scatterplot of regional PLS1 scores (weighted sum of 20,647 gene expression scores) vs. case–control differences in regional morphometric similarity (Cobre dataset). (*B*) Cortical map of regional PLS1 scores. (*C*) Cortical map of mean case–control morphometric similarity differences averaged across all datasets. Here, we include intrahemispheric left hemisphere edges only (*Materials and Methods*). (*D*) Genes that are strongly positively weighted on PLS1 (e.g., *LYSMD4*) correlate positively with case–control differences in regional morphometric similarity (r=0.44, P<0.001), whereas genes that are strongly negatively weighted on PLS1 (e.g., *C1orf95*) correlate negatively with case–control differences in morphometric similarity (r=−0.37, P<0.001).

We used PLS regression to identify patterns of gene expression that were correlated with the anatomical distribution of case–control morphometric similarity differences. The first PLS component explained 13% of the variance in the case–control morphometric similarity differences combining data from all three studies, significantly more than expected by chance (permutation test, P<0.001). PLS1 gene expression weights were positively correlated with case–control morphometric similarity differences in the Dublin study (r=0.49, P<0.001) and the Cobre study (r=0.37, P<0.001) (Fig. 2*A*) but not in the Maastricht study (r=0.006, P=0.94). These positive correlations mean that genes positively weighted on PLS1 are overexpressed in regions where morphometric similarity was increased in patients, while negatively weighted genes are overexpressed in regions where morphometric similarity was decreased in patients (Fig. 2*D*). Hence, genes that are positively (or negatively) weighted on PLS1 were related to increased (or decreased) morphometric similarity in cases compared with controls.

### Enrichment Analysis of Genes Transcriptionally Related to Morphometric Similarity.

We found 1,110 genes with normalized PLS1 weights Z<−3, which we denote the PLS− gene set, and 1,979 genes with Z>3, which we denote the PLS+ gene set. We first consider PLS− genes (the equivalent results for PLS+ genes are also given below).

**Fig. 3.**
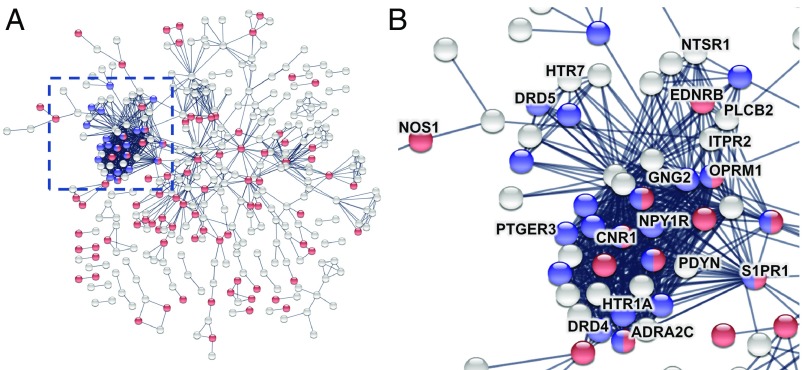
Enrichment analysis of genes transcriptionally related to morphometric similarity. (*A*) PPI network for PLS− genes (Z<−3) highlighted with some of the significantly GO-enriched biological processes: nervous system development in red and adenylate cyclase-modulating GPCR signaling pathway in blue. The most interconnected set of proteins was coded by several genes previously implicated in schizophrenia (highlighted in *B*; details are in the text and *SI Appendix*, section S8.8).

We mapped the network of known interactions between proteins coded by the PLS− gene set (14) (Fig. 3). The resulting protein–protein interaction (PPI) network had 341 connected proteins and 1,022 edges, significantly more than the 802 edges expected by chance (permutation test, $P<1^{-13}$). We also tested the PLS− gene set for significant gene ontology (GO) enrichment of biological processes and enrichment of Kyoto Encyclopedia of Genes and Genomes (KEGG) pathways. Enriched biological processes included “nervous system development,” “synaptic signaling,” and “adenylate cyclase-modulating G-protein coupled receptor (GPCR) signaling pathway” (Dataset S1). There were two significantly enriched KEGG pathways: “neuroactive ligand-receptor interaction” and “retrograde endocannabinoid signaling” (*SI Appendix*, Fig. S13). The proteins coded by genes enriched for adenylate cyclase-modulating GPCR signaling pathway and the two KEGG pathways formed the most strongly interconnected cluster of nodes in the PPI network (Fig. 3), compatible with them sharing a specialized functional role for GPCR signaling.

Genes recently reported as overexpressed in post mortem brain tissue from patients with schizophrenia (15) were highly enriched among genes that were negatively weighted on PLS1 (permutation test, P<0.001 after FDR correction). The relationship between the sign of PLS1 weights of gene expression related to the MRI case–control phenotype, and the sign of case–control differences in the histological measures of brain gene expression, was highly nonrandom (Wilcoxon rank sum test, *P* $<10^{-26}$).

In other words, genes that were up-regulated in post mortem brain tissue from patients with schizophrenia are normally overexpressed in association cortical areas that have reduced morphometric similarity in psychosis. This association between gene expression in regions with reduced morphometric similarity and genes up-regulated in schizophrenia was replicated by analysis of two alternative datasets provided by the PsychENCODE consortium (16) and in ref. 17 (*SI Appendix*, section S8.5). We also observed enrichment by genes up-regulated in other psychiatric disorders (e.g., autistic spectrum disorders), which is compatible with the substantial overlap between genes that are up-regulated (or down-regulated) in common between schizophrenia and other neurodevelopmental disorders (16).

The PLS+ genes coded proteins that formed a PPI network with significantly more edges than expected by chance (*P* $<10^{-6}$), which was enriched for the biological process “nucleic acid metabolic process” but no KEGG pathways (*SI Appendix*, Fig. S14). Genes that are down-regulated post mortem in schizophrenia (15) were highly enriched among genes that were positively weighted on PLS1 (permutation test, P<0.001 after FDR correction). This result was reproduced with genes reported as down-regulated in schizophrenia in ref. 17, although not by the PsychENCODE consortium (16) (*SI Appendix*, section S8.5).

There was no significant enrichment of PLS− or PLS+ genes for common sequence variants associated with schizophrenia derived from a recent genome-wide association study (GWAS) of Psychiatric Genomics Consortium (PGC) and CLOZUK samples (18) (P>0.05).

## Discussion

### Morphometric Similarity Network Phenotypes.

Morphometric similarity mapping disclosed a robust and replicable cortical pattern of differences in psychosis patients. Morphometric similarity was significantly reduced in frontal and temporal cortical areas and significantly increased in parietal cortical areas. This pattern was consistent across three independent datasets with different samples, locations, scanners, and scanning parameters.

What does this MRI phenotype of psychosis represent? Morphometric similarity quantifies the correspondence or kinship of two cortical areas in terms of multiple macrostructural features (e.g., cortical thickness) and microstructural features [e.g., fractional anisotropy (FA)] that are measurable by MRI. We assume that high morphometric similarity between a pair of cortical regions indicates that there is a high degree of correspondence between them in terms of cytoarchitectonic and myeloarchitectonic features that we cannot directly observe given the limited spatial resolution and cellular specificity of MRI. Prior work also showed that morphometrically similar cortical regions are more likely to be axonally connected to each other (i.e., morphometric similarity is a proxy marker for anatomical connectivity) (3). We, therefore, interpret the reduced morphometric similarity that we observe in frontal and temporal brain regions in psychosis as indicating that there is reduced architectonic similarity or greater architectonic differentiation between these areas and the rest of the cortex, which is probably indicative of reduced anatomical connectivity to and from the less similar, more differentiated cortical areas.

There is a well-evidenced and articulated prior theory of schizophrenia as a dysconnectivity syndrome; specifically, functional dysconnectivity of frontal and temporal cortical areas has been recognized as a marker of brain network disorganization in schizophrenia (19). Our results of reduced morphometric similarity in frontal and temporal cortices—implying increased architectonic differentiation and decreased axonal connectivity—are descriptively consistent with this theory. Our complementary finding of abnormally increased morphometric similarity in parietal cortex—implying increased architectonic similarity and axonal connectivity—is plausible but not so clearly precedented given the relatively limited prior data on the parietal cortex in studies of schizophrenia as a dysconnectivity syndrome (20, 21).

Encouragingly, this MRI network marker of psychosis was highly reliable across three independent and methodologically various case–control studies. This implies that the measurement is robust enough to be plausible as a candidate imaging biomarker of cortical network organization in large-scale, multicenter studies of psychosis.

### Transcriptional Profiling of Morphometric Similarity Network Phenotypes.

In an effort to connect these MRI phenotypes to the emerging genetics and functional genomics of schizophrenia, we first used PLS to identify the weighted combination of genes in the whole genome that has a cortical expression map most similar to the cortical map of case–control morphometric similarity differences. Then, we tested the mechanistic hypothesis that the genes with greatest (positive or negative) weight on PLS1 were enriched for genes previously implicated in the pathogenesis of schizophrenia.

We found that the genes that are normally overexpressed in frontal and temporal areas of reduced morphometric similarity in psychosis were significantly enriched for genes that are up-regulated in post mortem brain tissue from patients with schizophrenia (15). Conversely, the genes that are normally overexpressed in parietal and other areas of increased morphometric similarity in psychosis were significantly enriched for genes that are down-regulated in post mortem data (15). This tight coupling between MRI-derived transcriptional weights and gene transcription measured histologically was highly significant and the association with up-regulated genes was replicated across three prior post mortem datasets.

Additional investigation showed that the proteins coded by the PLS− genes formed a dense, topologically clustered interaction network that was significantly enriched for a number of relevant GO biological processes and KEGG pathways. The cluster of interactive proteins related to GPCR signaling included multiple proteins coded by genes previously linked to antipsychotic mechanisms of action [including *DRD4* (22), *HTR1* (23), *NTSR1* (24), and *ADRA2C* (25)], reported in transcriptional studies of post mortem brain tissue [e.g., *PTGER3*, *S1PR1*, *ITPR2*, and *EDNRB* (15, 26)], or associated with risk SNPs for schizophrenia [e.g., *DRD5*, *OPRM1*, and *CNR1* (27–29)]. The remarkable density of therapeutically relevant genes in the GPCR-related cluster suggests that other topologically neighboring genes may deserve additional attention as targets for antipsychotic interventions.

Risk genes identified by the largest extant GWAS of schizophrenia were not significantly enriched among PLS− or PLS+ genes. Nevertheless, the involvement of PLS− genes farther down the causal pathway is still mechanistically revealing and potentially useful.

### Methodological Considerations.

Some limitations of this study should be highlighted. The whole-brain data on “normal” brain tissue expression of the genome were measured post mortem in six adult brains (mean age = 43 y) and not in age-matched subjects or patients with schizophrenia (such data are not currently available to our knowledge). Also, the transcriptional experiments that we use to label genes as up- or down-regulated in schizophrenia were performed in regions of the parietal or prefrontal cortex (15), whereas the neuroimaging results are for the whole brain. We have used MRI data from three independent studies to measure morphometric similarity networks, but the studies used different scanning protocols, leading to estimation of morphometric similarity between regions on the basis of seven MRI parameters that were measurable in all studies. Future work could usefully explore the opportunity to further improve sensitivity and reliability of the morphometric similarity network biomarker of schizophrenia by optimizing and standardizing the MRI procedures to measure the most informative set of morphometric features. Finally, the datasets have varied, limited clinical information available, making it difficult to assess the clinical significance of the morphometric similarity phenotype.

## Materials and Methods

### Samples.

We used MRI data from three prior case–control studies: the Maastricht GROUP study (30) from The Netherlands; the Dublin dataset, which was acquired and scanned at the Trinity College Institute of Neuroscience as part of a Science Foundation Ireland-funded neuroimaging genetics study (“A structural and functional MRI investigation of genetics, cognition and emotion in schizophrenia”); and the publicly available Cobre dataset (31). The Maastricht and Dublin datasets were PSYSCAN legacy datasets. The standing ethics committee of Maastricht University Medical Center approved the Maastricht GROUP study. The St. James Hospital and the Adelaide and Meath Hospital Dublin Incorporating the National Children’s Hospital (AMNCH) joint ethics boards approved the Dublin study. All participants gave informed consent. All patients satisfied *Diagnostic and Statistical Manual of Mental Disorders IV* (DSM-IV) diagnostic criteria for schizophrenia or other nonaffective psychotic disorders. MRI data were quality controlled for motion artifacts (*SI Appendix*, section S1). The Euler number, which quantifies image quality (32), was not significantly different between groups in any of the studies, but it was different between studies, indicating that the studies were ranked Dublin > Cobre > Maastricht in terms of image quality (*SI Appendix*, Table S1).

### Morphometric Similarity Mapping.

The T1-weighted MRI data [magnetization-prepared rapid gradient-echo (MPRAGE) sequence] and the DWI data from all participants were preprocessed using a previously defined computational pipeline (6). Briefly, we used the recon-all (33) and trac-all (34) commands from FreeSurfer (version 6.0). Following ref. 3, the surfaces were then parcellated using an atlas with 308 cortical regions derived from the Desikan–Killiany atlas (6, 35). For each region, we estimated seven parameters from the MRI and DWI data: gray matter volume, surface area, cortical thickness, Gaussian curvature, mean curvature, FA, and mean diffusivity. Each parameter was normalized for sample mean and SD before estimation of Pearson’s correlation for each pair of *Z*-scored morphometric feature vectors, which were compiled to form a 308×308 morphometric similarity matrix $M_{i}$ for each participant, i=1,…N (3).

### Case–Control Analysis of Morphometric Similarity Networks.

The global mean morphometric similarity for each participant is the average of $M_{i}$. The regional mean $MS_{i,j}$ for the ith participant at each region j=1,…,308 is the average of the jth row (or column) of $M_{i}$. Thus regional MS strength is equivalent to the weighted degree or hubness of each regional node, connected by signed and weighted edges of pair-wise similarity to all other nodes in the whole brain connectome represented by the morphometric similarity matrix. For global and regional morphometric similarity statistics alike, we fit linear models to estimate case–control difference, with age, sex, and age × sex as covariates. Our main results also replicated in subsets of the data balanced for age and sex (*SI Appendix*, section S5.6). *P* values for case–control differences in regional morphometric similarity were combined across all three studies using Fisher’s method. The resulting *P* value for each region was thresholded for significance using FDR<0.05, to control type 1 error over multiple (308) tests.

### Transcriptomic Analysis.

We used the AHBA transcriptomic dataset with gene expression measurements in six post mortem adult brains (36) (human.brain-map.org) ages 24–57 y. Each tissue sample was assigned to an anatomical structure using the AHBA MRI data for each donor (37). Samples were pooled between bilaterally homologous cortical areas. Regional expression levels for each gene were compiled to form a 308×20,647 regional transcription matrix (37). Since the AHBA only includes data for the right hemisphere for two subjects, in our analyses relating gene expression to MRI data, we only consider intrahemispheric left hemisphere edges (38).

We used PLS to relate the regional morphometric similarity case–control differences (t statistics from the 152 cortical regions in the left hemisphere calculated from intrahemispheric edges only) to the post mortem gene expression measurements for all 20, 647 genes. PLS uses the gene expression measurements (the predictor variables) to predict the regional morphometric similarity case-control t statistics from all three datasets (the response variables). The first PLS component (PLS1) is the linear combination of the weighted gene expression scores that have a cortical expression map that is most strongly correlated with the map of case–control morphometric similarity differences. The statistical significance of the variance explained by PLS1 was tested by permuting the response variables 1,000 times. The error in estimating each gene’s PLS1 weight was assessed by bootstrapping (resampling with replacement of the 308 cortical regions), and the ratio of the weight of each gene to its bootstrap SE was used to calculate the *Z* scores and, hence, rank the genes according to their contribution to PLS1 (6).

We constructed PPI networks from the genes with PLS1 weights Z>3 and Z<−3 (all FDR<0.05) using STRING version 10.5 (14). Our key results were robust to changing these thresholds to Z>4 and Z<−4 (all FDR<0.01) (*SI Appendix*, section S8.3). We used DAVID (39, 40) to calculate enrichments of KEGG pathways and GO enrichments of biological processes for genes with Z>3 or Z<−3 using a background gene list of 15,745 brain-expressed genes (*SI Appendix*, section S8.3) (38).

We used a resampling procedure to test for enrichment of PLS-derived gene sets by genes previously associated with schizophrenia by transcriptional data (15). The median rank of each risk gene set in the PLS gene list was compared with the median rank of 10,000 randomly selected brain-expressed gene sets (3).

### Data and Code Availability.

The data used for the analyses are available at https://doi.org/10.6084/m9.figshare.7908488.v1 (5) and the code can be found at https://github.com/SarahMorgan/Morphometric_Similarity_SZ (41).

## Supplementary Material

Supplementary File

Supplementary File

Supplementary File

Supplementary File

Supplementary File
