# Supplementary material for: Cortical patterning of abnormal morphometric similarity in psychosis is associated with brain expression of schizophrenia-related genes
Source: Proc Natl Acad Sci U S A. 2019 Apr 19;116(19):9604–9. doi: 10.1073/pnas.1820754116 (PMC6511038; doi:10.1073/pnas.1820754116)
Supplement: Supplementary File [file pnas.1820754116.sapp.pdf]

Supplementary Information for "Cortical patterning of abnormal morphometric similarity in psychosis is associated with brain expression of schizophrenia related genes"

Sarah E Morgan, Jakob Seidlitz, Kirstie J Whitaker, Rafael Romero-Garcia, Nicholas E Clifton, Cristina Scarpazza, Therese van Amelsvoort, Machteld Marcelis, Jim van Os, Gary Donohoe, David Mothersill, Aiden Corvin, Andrew Pocklington, Armin Raznahan, Philip McGuire, Petra E Vértes*, Edward T Bullmore*

March 7, 2019

Contents

1 Datasets 2
1.1 Maastricht GROUP . . . 2
1.2 Dublin . . . 2
1.3 Cobre . . . 2

2 Motion 4

3 Global case-control MS differences 6

4 Regional case-control MS differences in individual datasets 6

5 Robustness of results 8
5.1 Permuting the group labels . . . 8
5.2 Correlation between case-control t-statistic and control MS . . . 8
5.3 Correlations between datasets . . . 9
5.4 Spatial permutation . . . 9
5.5 Effect sizes . . . 9
5.6 Sex and age matched analyses . . . 10
5.7 Outlier . . . 11

6 Symptoms 11
6.1 Convert Dublin dataset symptoms to PANSS . . . 11
6.2 Excluding Maastricht patients with low symptom scores . . . 12
6.3 Relationship between MS and symptoms and other clinical/functional measures . . . 13

7 Yeo networks and von Economo classes 17

8 Transcriptomic analysis 18
8.1 Left hemisphere t-statistics . . . 18
8.2 Spatial permutation test- correlation between PLS1 and t-statistics . . . 18
8.3 PPI network analysis . . . 18
8.3.1 PPI network from genes with $Z < -3$ . . . 19
8.3.2 PPI network from genes with $Z > 3$ . . . 19
8.3.3 PPI networks with $Z > 4$ and $Z < -4$ . . . 19

*PEV and ETB contributed equally to the work.

www.pnas.org/cgi/doi/10.1073/pnas.1820754116

8.4 Gandal gene enrichments and specificity . . . 24
8.5 PsychENCODE and Fromer gene enrichments . . . 24
8.6 GAD . . . 25
8.7 GWAS analyses . . . 25
8.8 Discussion of GPCR gene cluster . . . 27
8.9 Yeo networks and von Economo classes . . . 27

1 Datasets

Further details of the three datasets are given below. The datasets were chosen because they had both T1w MPRAGE and DWI images from 3T scanners for large (N > 100) sample sizes. In all three datasets, only subjects with both DWI and T1w images were included. Details of the previously defined computational pipeline used to pre-process the data are given in [1, 2]. Table S1 gives the data demographics.

1.1 Maastricht GROUP

The Maastricht GROUP dataset comes from an MRI study in Maastricht, the Netherlands, led by the GROUP consortium. Patients were identified by screening caseloads of representative clinicians for inclusion criteria in selected representative geographic areas of the Netherlands and Belgium. The data was acquired using a 3T Siemens Magnetom Allegra head scanner. For more information, see [3]. 2 patients were excluded due to movement artifacts based on visual QC of the T1w data, leaving 83 patients and 68 control subjects. The diagnoses of the patients are heterogeneous and includes 57 patients with schizophrenia, 11 patients with psychotic disorder, 2 patients with brief psychotic disorder, 9 patients with schizoaffective disorder and 4 patients with schizophreniform disorder.

1.2 Dublin

The Dublin dataset was acquired and scanned in the Trinity College Institute of Neuroscience as part of a Science Foundation Ireland-funded neuroimaging genetics study ("A structural and functional MRI investigation of genetics, cognition and emotion in schizophrenia"). Patients were recruited through local clinical services whilst healthy control subjects reported no history of psychiatric disease. Both groups were recruited in the same geographical area through local advertisement and exclusion criteria for both groups included confirmed or suspected pregnancy, any history of neurological disorders or intellectual disability and substance misuse in the preceding 3 months. The data was acquired using a 3T Philips Intera Achieva scanner. 5 patients and 4 control subjects were excluded from the Dublin dataset due to movement artifacts based on visual QC of the T1w data. A large number of subjects also had to be excluded due to poor quality of DTI which led to failure to pass the pre-processing pipeline described below (51 control subjects and 9 patients). 82 control subjects and 33 patients remained, of whom 3 were diagnosed with schizoaffective disorder and 30 with schizophrenia.

1.3 Cobre

The Cobre dataset was downloaded from the SchizConnect database (http://schizconnect.org), where it had been obtained from the COllaborative Informatics and Neuroimaging Suite Data Exchange tool (COINS; http://coins.mrn.org/dx). In this dataset, a diagnosis of schizophrenia was made using the Structured Clinical Interview for DSM Disorders (SCID; Diagnostic and Statistical Manual of Mental Disorders, DSM-IV). Exclusion criteria included confirmed or suspected pregnancy, any history of neurological disorders and a history of mental retardation. The data was acquired using a 3T Siemens scanner. 60 patients were diagnosed with schizophrenia and 9 with schizoaffective disorder.

	Maastricht GROUP		Dublin		Cobre	
	CON	PAT	CON	PAT	CON	PAT
Sample size	68	83	82	33	77	69
Age (years)	29.4 ± 10.3	28.4 ± 7.0	33.5 ± 12.6	42.2 ± 11.7	38.0 ± 12.0	38.2 ± 13.3
Sex (M)	28 (41.2%)	56 (67.5%)	35 (42.7%)	24 (72.7%)	58 (75.3%)	55 (77.5%)
PANSS total	31.8 ± 4.3*	42.8 ± 12.7*	N/A	N/A	N/A	60.5 ± 16.6*
PANSS positive	7.4 ± 1.1*	9.5 ± 3.7*	N/A	N/A	N/A	15.3 ± 5.1*
PANSS negative	7.2 ± 0.9*	10.5 ± 5.1*	N/A	N/A	N/A	15.2 ± 5.3*
PANSS general	17.3 ± 2.7*	23.0 ± 6.1*	N/A	N/A	N/A	30.1 ± 9.9*
SAPS (composite)	N/A	N/A	N/A	12.9 ± 16.4*	N/A	N/A
SANS (composite)	N/A	N/A	N/A	16.0 ± 16.5*	N/A	N/A
Euler number	-95.7 ± 49.0	-96.5 ± 53.9	-35.5 ± 18.7	-30.9 ± 19.6	-66.6 ± 36.4	-66.0 ± 31.0

Table S1: Maastricht GROUP, Dublin and Cobre dataset demographics (after after subjects with poor data quality were excluded). *Dublin symptom scores were available for 24 patients only. Cobre symptom scores were available for 67 patients. Maastricht symptom scores were available for 63 control subjects and 80 patients.

2 Motion

To check for differences in motion and image quality between the patients and the control subjects we calculate the Euler number for each T1w image. This approach was proposed by [4] as a way to quantitatively assess image quality. Using a two-sided t-test we find no significantly significant differences in the Euler number between the two groups in any of the three datasets, as shown in Figure S1. We note that there are some differences in Euler number between datasets- for Maastricht the mean Euler number and standard deviation is -96.2 ± 48.9, for Dublin we obtain -34.3 ± 18.9 and for Cobre we obtain -66.3 ± 33.8. Tentatively, this would suggest that the data quality is highest for the Dublin dataset and lowest for the Maastricht dataset, with the Cobre dataset in between the two. The exact extent to which the Euler number can be compared between datasets obtained using different scanners is unclear, for example [4] found that when predicting manually assessed image quality from the Euler number the most accurate classifications were obtained when classification threshold was allowed to vary by dataset, although accuracies of above 75% could still be obtained using a fixed threshold. Further work is needed in this area.

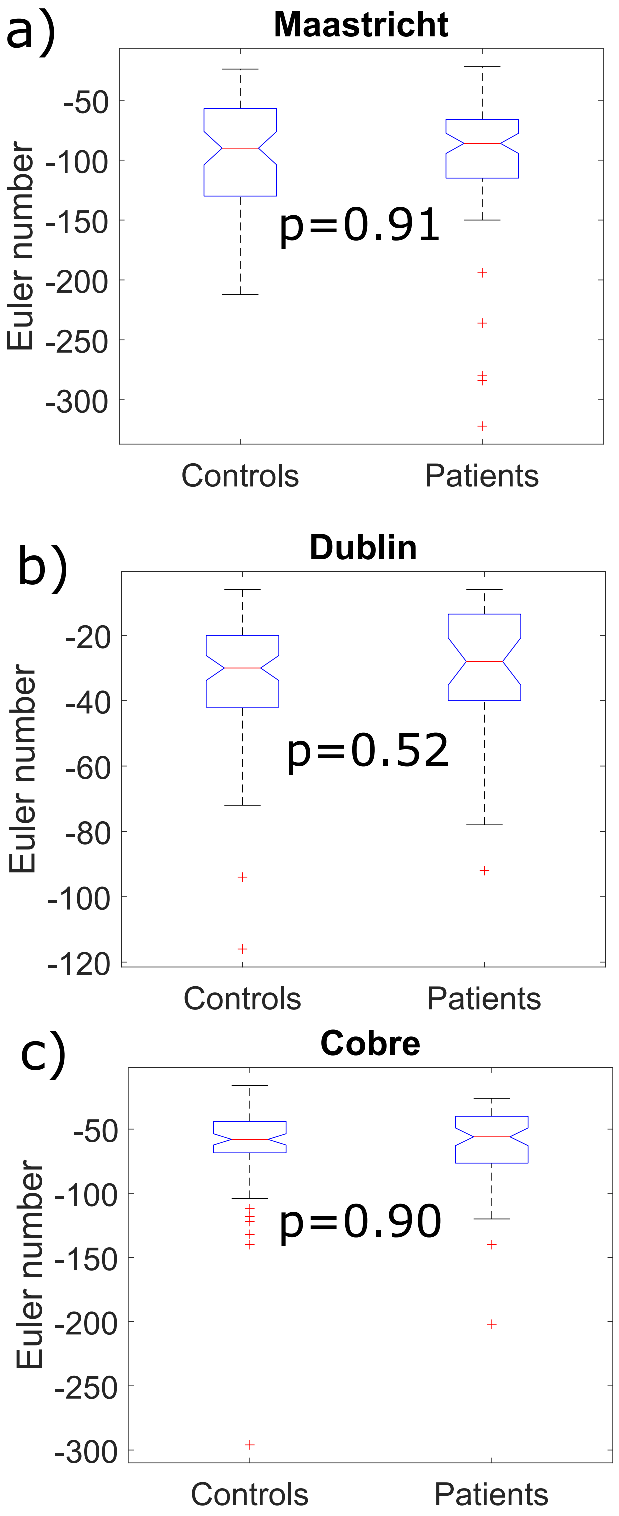

Figure S1: Box plots for the Euler number in the patient and control groups, for all three datasets.

3 Global case-control MS differences

Figure S2 plots the global mean and distributions of regional morphometric similarity in all three datasets.

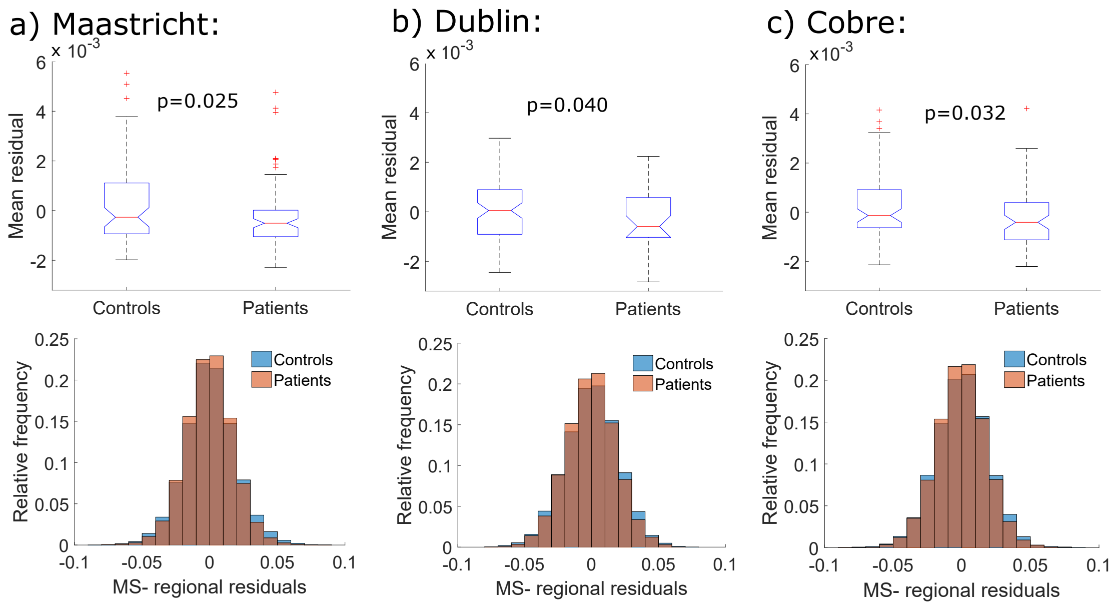

Figure S2: **Global mean and distributions of regional morphometric similarity.** Top panel: Box plots for global mean MS (after regressing sex and age) in controls and patients, in each of the three datasets (Maastricht, Dublin, COBRE). Bottom panel: Distributions of regional similarity strength, i.e., the average similarity of each region with all other regions, after regressing age and sex, for controls (blue) and patients (red), in each of the three datasets.

4 Regional case-control MS differences in individual datasets

Figure S3 shows the regional patient/control MS t-statistics from each dataset, with regions where $P < 0.05$ only. Note that these results are shown before correcting for the false discovery rate, which is performed after combining the datasets. There are 90 significant regions in the Dublin dataset, 37 in the Cobre dataset and 30 in the Maastricht dataset, suggesting that the strongest signal is in the Dublin dataset.

In Figure S4, we plot the correlations between the t-statistics from the three different datasets, pairwise. As noted in the text, we find that the Dublin t-statistic is positively correlated with both the Maaastricht t-statistic (r=0.42, p< 0.001) and the Cobre t-statistic (r=0.47, p< 0.001), although the Maastricht and Cobre t-statistics are not correlated with each other (r=0.058, p=0.31).

After combining the datasets and correcting for FDR, 18 regions show statistically significant MS differences between control subjects and patients. The regions are listed in Table S2.

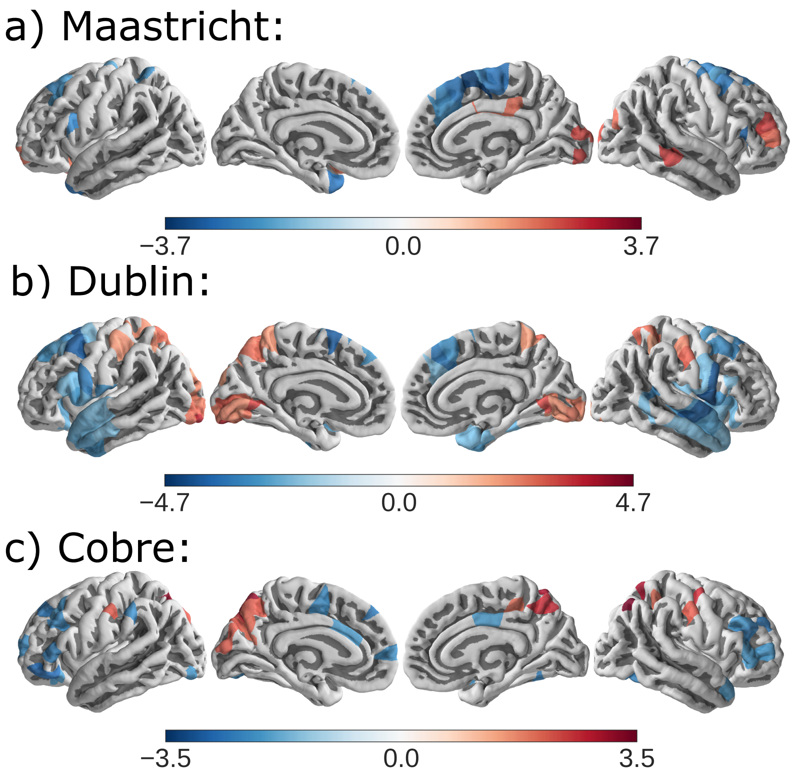

Figure S3: Patient/control MS t-statistics from a) Maastricht, b) Dublin and c) Cobre, with regions where $p < 0.05$ only, before FDR correction.

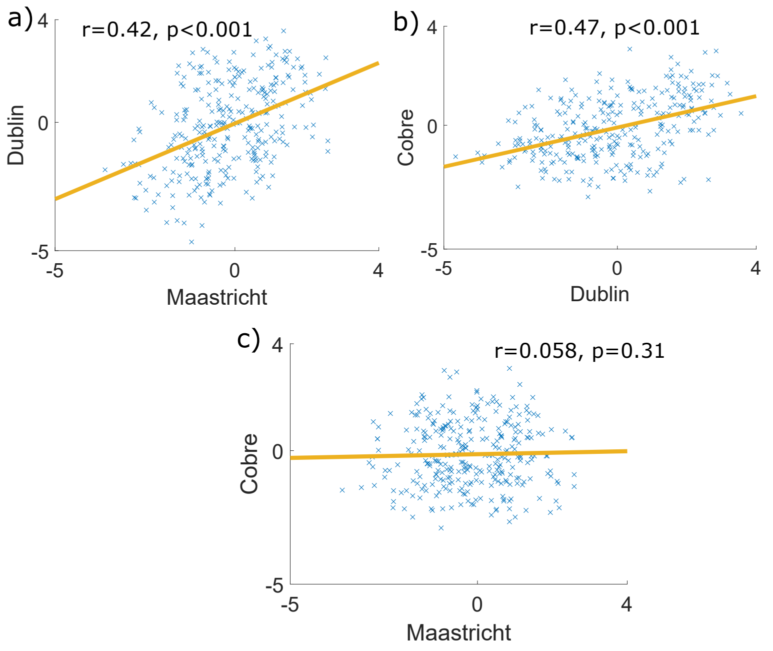

Figure S4: Correlations between the t-statistics from a) Dublin and Maastricht, b) Dublin and Cobre and c) Maastricht and Cobre.

Name	x	y	z	Control MS	Patient MS	t-statistic	P-value
lh_caudalmiddlefrontal_part1	-33.27	20.20	45.35	0.024	0.016	-2.19	0.035
lh_caudalmiddlefrontal_part4	-39.72	11.34	48.85	0.022	0.013	-2.27	0.015
lh_precentral_part4	-54.64	3.80	21.88	0.023	0.015	-2.31	0.015
lh_superiorfrontal_part8	-10.01	10.76	61.43	0.012	0.002	-2.27	0.015
lh_superiorfrontal_part9	-12.80	41.99	41.76	0.028	0.021	-2.38	0.015
lh_superiorfrontal_part11	-17.72	30.84	48.03	0.021	0.011	-2.02	0.049
lh_superiorfrontal_part12	-17.76	21.07	55.61	0.017	0.006	-2.33	0.015
lh_superiorparietal_part10	-12.30	-70.56	52.29	-0.014	-0.007	1.72	0.022
rh_caudalmiddlefrontal_part4	36.44	12.22	48.85	0.016	0.007	-2.43	0.015
rh_parstriangularis_part2	43.06	25.40	5.94	0.001	-0.008	-2.11	0.035
rh_postcentral_part6	54.62	-14.84	36.20	-0.018	-0.011	2.01	0.049
rh_precentral_part1	50.42	1.97	8.00	0.026	0.017	-2.42	0.014
rh_precentral_part3	54.26	5.64	24.20	0.019	0.010	-2.45	0.015
rh_superiorfrontal_part7	9.69	8.29	60.03	0.014	0.005	-2.36	0.015
rh_superiorfrontal_part11	9.20	24.39	53.69	0.020	0.012	-2.18	0.019
rh_superiorfrontal_part13	9.66	30.31	45.21	0.027	0.018	-2.29	0.015
rh_superiorparietal_part9	18.52	-59.15	61.76	-0.010	-0.004	1.68	0.015
rh_superiortemporal_part4	56.76	-7.72	-6.31	0.026	0.018	-2.05	0.018

Table S2: Table giving details for the 18 statistically significant regions- anatomical labels, coordinates (in fsaverage, MNI305 space), mean MS value in control subjects and patients (averaged across datasets), mean t-statistic (averaged across datasets) and P-value after FDR correction.

5 Robustness of results

5.1 Permuting the group labels

To assess the robustness of the regional MS results, we re-calculated the t-statistic for control/patient differences 1000 times with scrambled group labels. For each region we then calculated a P-value to assess whether the empirically calculated regional t-statistic was greater than the values from scrambled group labels (or less than in the case of a negative empirical t-statistic). Regions with significant P-values after combining the datasets using Fisher's method are shown in Figure S5.

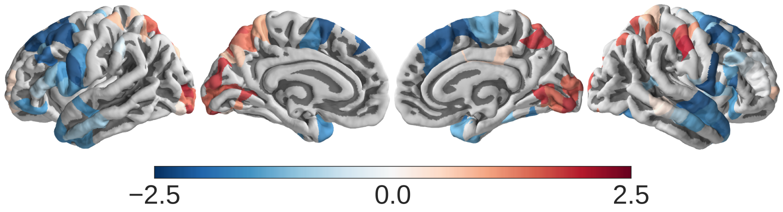

Figure S5: Regions with significant P-values across the three datasets in comparison to the P-values obtained with scrambled group labels.

5.2 Correlation between case-control t-statistic and control MS

In the main text we showed that there was a negative correlation between the case/control regional differences in MS (the mean t-statistic) and the regional pattern of MS in control subjects (Fig. 1f). To assess the robustness of this correlation, we performed a permutation test, where we averaged the randomised t-statistics calculated above (by scrambling the group labels) across datasets and then correlated the resulting mean randomised t-statistics with the mean control MS pattern. We obtain $P_{perm} = 0.002$, hence the correlation shown in Fig. 1f is not expected by chance if the group labels are permuted.

5.3 Correlations between datasets

In order to assess the robustness of the correlations between datasets, we correlate the 1000 randomised t-statistics (obtained using scrambled group labels) pairwise between datasets. We then calculate a P-value based on the number of times the randomised correlations are greater than the real correlations observed between the datasets (a one-sided test). The results are given in Table S3. We find that the statistically significant correlations between the Maastricht and Dublin datasets and the Cobre and Dublin datasets reported in the main text are remarkably robust to this randomisation.

Datasets	Pearson r	Pearson P-value	Permutation test P-value
Maastricht/Dublin	0.42	< 0.001	0.0050
Maastricht/Cobre	0.058	0.31	0.36
Dublin/Cobre	0.47	< 0.001	< 0.001

Table S3: Pearson correlation values (r) for the correlations between t-statistics from the three datasets, alongside the corresponding Pearson correlation P-value and a P-value calculated using permuted group labels.

5.4 Spatial permutation

Finally, the correlations between the regional t-statistics from different datasets reported in the main text assume that the number of samples is equal to the number of regions, which is not the case because the number of regions is arbitrary (due to the resolution of the chosen parcellation) and non-independent (due to spatial autocorrelation amongst neighboring parcels). To address this issue, we use a regional spatial permutation test, as proposed by [5]. Here the idea is to compare the empirical correlation between the two t-statistics to null correlations generated by randomly rotating the spherical projection of one of the two spatial maps (as generated in FreeSurfer), before projecting it back onto the brain surface. The rotated projection preserves both the spatial contiguity and the hemispheric symmetry of the empirical maps. See [5] for more details. The results are given in Table S4 and show that the correlations between the Maastricht GROUP and Dublin datasets and between the Dublin and Cobre datasets are robust to controlling for these effects.

Datasets	Pearson r	Pearson P-value	Spatial permutation P-value
Maastricht/Dublin	0.42	< 0.001	< 0.001
Maastricht/Cobre	0.058	0.31	0.38
Dublin/Cobre	0.47	< 0.001	< 0.001

Table S4: Pearson correlation values (r) for the correlations between t-statistics from the three datasets, alongside the corresponding Pearson correlation P-value and a P-value calculated using spatial permutation.

5.5 Effect sizes

To assess the size of the global and regional differences in morphometric similarity, we used Hedges's g, which is an unbiased version of Cohen's d, and describes the standardized mean difference of an effect [6]. We calculated Hedge's g directly from the t-statistics, as described by [6]. The regional results are shown in Figure 1 of the main text. The largest positive and negative regional effect sizes in the three datasets are given in Table S5, alongside the effect size for the global reduction in MS in patients. The regional results suggest medium to large effect sizes in the parts of the cortex which show the largest differences in MS (in frontal, temporal and parietal areas), according to the criteria proposed by [7] (where 0.2 is a small effect size, 0.5 is a medium effect size and 0.8 is a large effect size). The global results suggest small-medium global effect sizes.

	Maastricht	Dublin	Cobre
Largest positive regional effect size	0.42	0.73	0.57
Largest negative regional effect size	-0.59	-0.95	-0.47
Effect size for global reduction in MS	-0.37	-0.42	-0.36

Table S5: Summary of Hedge's g effect sizes in Maastricht, Dublin and Cobre.

5.6 Sex and age matched analyses

Both the Maastricht and Dublin datasets exhibited a group difference in sex, and the Dublin dataset also exhibited a group difference in age. Whilst we controlled for age and sex in all of our analyses in the main text, here we show that our main results also replicated in subsets of the data balanced for age and sex. In particular, we recalculated our main results first for the male subjects only in both the Maastricht and Dublin datasets and second for age-matched subjects in the Dublin dataset. For the first analysis, we took the male subjects because there were more male subjects than female subjects. In the latter analysis, we matched each patient to a control subject of the same age (the same number of years old). This was possible for 24 out of the 33 patients, giving a total sample size of 24 patients and 24 healthy controls (48 subjects in total).

The results are shown in Figures S6 and S7. In both Maastricht and Dublin, there is a significant decrease in MS in male patients compared to male healthy control subjects, confirming our previous results ($P = 0.011$ in Maastricht and $P = 0.011$ in Dublin). The t-statistics calculated from the male subjects only correlate strongly with the t-statistics reported in the main text from the whole cohort (regressing age and sex) (Pearson correlation coefficient r=0.84, $P < 0.001$ in Maastricht and r=0.94, $P < 0.001$ in Dublin). Similarly, there was a decrease in MS in the age-matched Dublin patients compared to the age-matched healthy control subjects ($P = 0.0080$), and the t-statistic calculated from the age-matched subjects correlated strongly with the t-statistic reported in the main text from the whole Dublin cohort (r=0.92, $P < 0.001$).

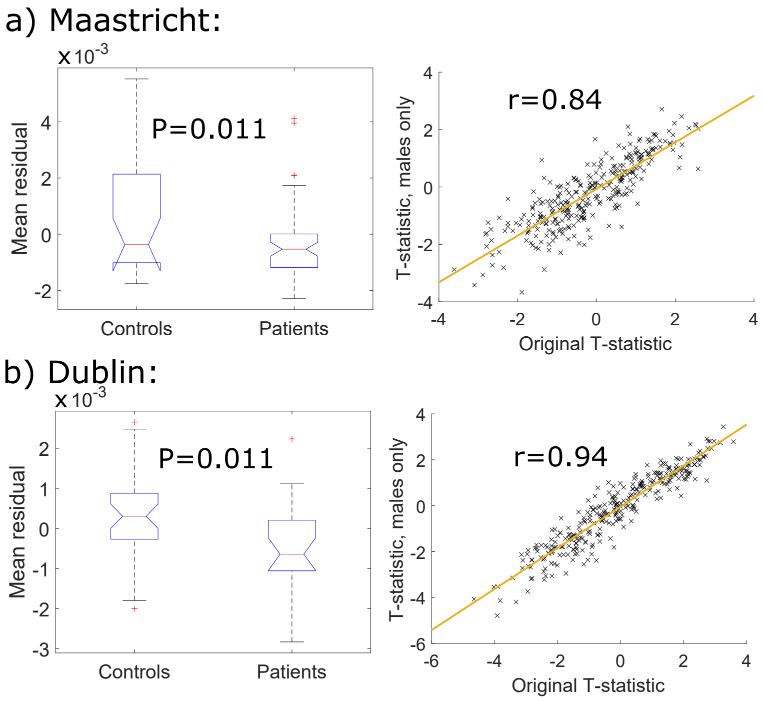

Figure S6: The global decrease in MS replicated with male subjects only in a) the Maastricht and b) the Dublin datasets. The original case/control t-statistics reported in the main text were also highly correlated with the t-statistics including male subjects only.

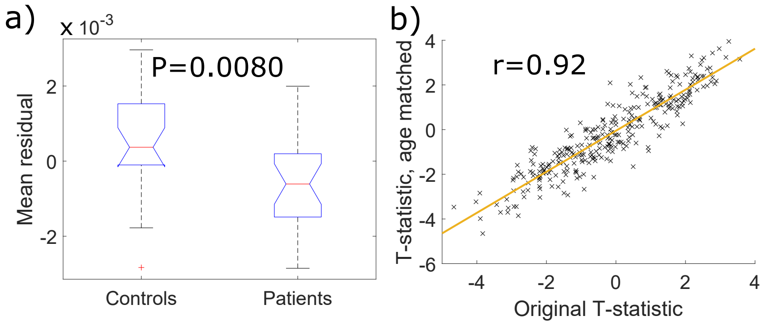

Figure S7: The global decrease in MS replicated with age-matched patients and controls in the Dublin dataset. The original case/control t-statistic reported in the main text was also highly correlated with the age-matched t-statistic.

5.7 Outlier

We note that there is one subject with outlier MD and FA values in the Cobre dataset. The data passed the pre-processing pipeline, however the output MD and FA values are substantially lower than the mean MD and FA values for the other subjects (more than 5 standard deviations), in all regions. This is the only subject for whom this is a problem, and there are no such subjects in either the Maastricht GROUP or Dublin datasets. If this subject is excluded from the analyses the results remain almost identical- the global morphometric similarity still decreases (p=0.034), as shown in Figure S8 b) and the t-statistics for the regional control/patient MS differences before and after excluding the subject correlate with Pearson correlation coefficient r=0.9973. This analysis confirms that the outlier subject does not drive our results and also suggests that our results are extremely robust to any individual subject.

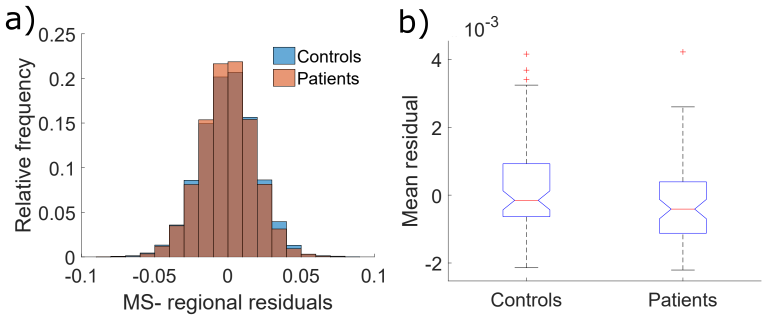

Figure S8: a) MS correlation distribution for Cobre with outlier subject excluded (see text for details). b) With the outlier subject excluded, mean MS is still reduced in patients compared to control subjects, as reported in the main text (p=0.034).

6 Symptoms

6.1 Convert Dublin dataset symptoms to PANSS

The Maastricht GROUP dataset and the Cobre dataset both report symptom information using the Positive and Negative Syndrome Scale for Schizophrenia (PANSS), whilst the Dublin dataset reports symptom information using the Scale for the Assessment of Positive Symptoms (SAPS) and the Scale for the Assessment of Negative Symptoms (SANS). [8] proposed formulae to convert between PANSS and SAPS/SANS measures:

$$\text{PANSS positive} = 11.1886 + (0.2587 \times \text{SAPS [Composite] Total score}) \quad \text{(S1)}$$

$$\text{PANSS negative} = 7.1196 + (0.3362 \times \text{SANS [Composite] Total score}) \quad \text{(S2)}$$

If these formulae are applied to the Dublin symptom scores shown in Table 1 of the main text, the Dublin PANSS positive score is 14.5 ± 4.2 and the Dublin PANSS negative score is 12.5 ± 5.5. Comparisons of symptom scores between datasets are given in Table S6. The results suggest that the Dublin and Cobre patients are more symptomatic than the patients from the Maastricht GROUP dataset in terms of positive symptom scores, whilst there is no statistically significant difference between the Dublin and Cobre datasets. The Cobre dataset is more symptomatic than either of the other two datasets in terms of negative symptom scores.

	PANSS positive	PANSS negative
Maastricht/Dublin	✓(p< 0.001)	✗(p=0.10)
Maastricht/Cobre	✓(p< 0.001)	✓(p< 0.001)
Dublin/Cobre	✗(p=0.51)	✓(p< 0.001)

Table S6: Results from 2-sided t-tests to check for significant differences in the positive/negative symptom measures between the different datasets.

6.2 Excluding Maastricht patients with low symptom scores

Figure S9 plots a histogram of the Maastricht patients' total PANSS scores. The minimum possible total PANSS score is 30, hence the Figure shows that many of the Maastricht patients have very low symptom scores.

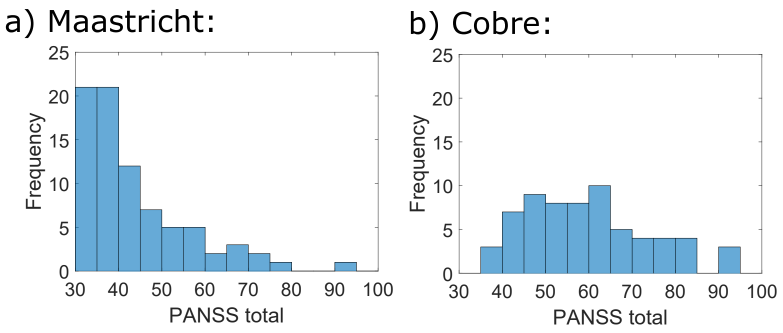

Figure S9: Histograms showing PANSS total symptom scores in a) Maastricht. b) PANSS total symptom scores in Cobre are also shown for comparison (PANSS total symptom scores were not available for Dublin and are therefore not shown).

To assess whether low symptom scores in the Maastricht study might be responsible for the lack of correlation between the case/control Maastricht and Cobre t-statistics, we re-calculated the Maastricht case/control t-statistic excluding patients with very low symptom scores. In particular, we excluded patients with PANSS total < 45, including only the N=26 Maastricht patients with PANSS total ≥ 45. We chose the threshold PANSS total $= 45$ based on work by [9], which showed that a PANSS total score of approximately corresponded to the CGI severity score 'borderline mentally ill', which is the first CGI severity score above 'normal'. Hence PANSS total < 45 was below the threshold to be considered even 'borderline mentally ill'.

The Maastricht t-statistic after patients with low symptom scores were excluded is plotted in Figure S10 and correlated significantly with the Cobre t-statistic (r=0.22, $P < 0.001$). The correlation with the Dublin t-statistic also increased, see Table S7. These results were robust to changing the threshold at which patients with low symptoms were excluded, for example we obtained very similar results when patients with PANSS total < 40 or PANSS total < 50 were excluded (namely significant correlations between the Maastricht and Cobre t-statistics, and increased correlation between the Maastricht and Dublin t-statistics).

To check that the increase in correlation between the Maastricht and Cobre datasets when excluding low symptom patients was not an artefact of reduced sample size, we performed a permutation test where instead of taking the 26 patients with PANSS total ≥ 45, the 26 patients were selected at random, 1000 times. We obtained $P_{perm} = 0.031$ for the significance of the correlation between the Maastricht and Cobre t-statistics when low symptom patients were excluded compared to random sets of excluded patients. In other words, the significant correlation of r=0.22 between

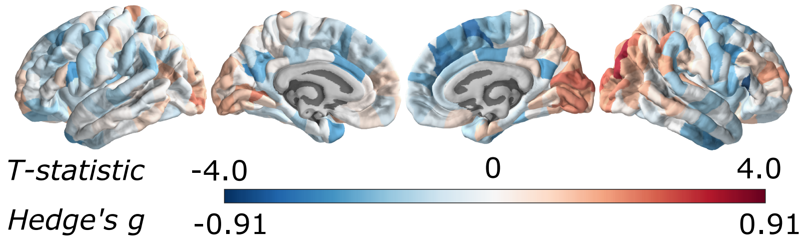

Figure S10: Maastricht t-statistic calculated excluding the subjects with PANSS total < 45.

	Maastricht *vs* Cobre t-statistics	Maastricht *vs* Dublin t-statistics
Before excluding patients with PANSS total $<$ 45 (as reported in the main text)	r=0.058, P=0.31	r=0.42, $P < 0.001$
After excluding patients with PANSS total < 45	r=0.22, $P < 0.001$	r=0.50, $P < 0.001$

Table S7: Correlations between Maastricht and Cobre or Dublin t-statistics, before and after excluding subjects with low symptom scores.

the Maastricht and Cobre t-statistics when low symptom patients were excluded was not expected when the same number of patients were excluded at random, suggesting that it was not an artefact of reduced sample size.

6.3 Relationship between MS and symptoms and other clinical/functional measures

To explore the clinical and functional significance of our results, we investigated the relationship between the MS results and the patients' symptom scores and other clinical/functional measures. We focused on the PANSS positive and negative scores, because they were available for all three datasets (rather than PANSS total scores which were not available in the Dublin dataset). For each patient, we calculated 1) the mean MS across all 308 cortical regions ($\overline{MS}_{global}$), 2) the mean MS across the 3 regions where MS increased significantly in patients compared to healthy control subjects (the red regions in the lower panel of Fig. 1e) ($\overline{MS}_{pos}$) and 3) the mean MS across the 15 regions where MS decreased significantly in patients (the blue regions in the lower panel of Fig. 1e) ($\overline{MS}_{neg}$). The latter two mesoscale measures were chosen to summarise the regional pattern of cortical differences in MS in patients compared to control subjects, see Fig. S11 for an illustration.

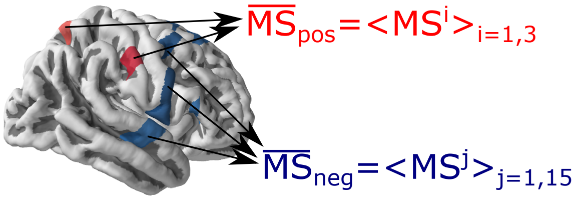

Figure S11: **Illustration of how $\overline{MS}_{pos}$ and $\overline{MS}_{neg}$ are calculated:** To calculate $\overline{MS}_{pos}$ for each patient, we average the patient's MS across the 3 regions where MS was significantly increased in patients compared to healthy control subjects. To calculate $\overline{MS}_{neg}$, we average MS across the 15 regions where MS was significantly decreased in patients compared to healthy control subjects.

To assess whether $\overline{MS}_{global}$, $\overline{MS}_{pos}$ and $\overline{MS}_{neg}$ were associated with symptoms, we fitted a linear model which used PANSS positive or PANSS negative scores to predict either $\overline{MS}_{global}$, $\overline{MS}_{pos}$ or $\overline{MS}_{neg}$, with age, sex, dataset and interaction terms as co-variates. We combined all datasets to obtain the maximum power possible. Table S8 shows the results. FDR multiple comparisons corrections were made for assessing the correlations with both PANSS positive and PANSS negative (2 comparisons).

	$\overline{MS}_{global}$			$\overline{MS}_{pos}$			$\overline{MS}_{neg}$		
Metric	t	P	P_{FDR}	t	P	P_{FDR}	t	P	P_{FDR}
PANSS positive	-1.16	0.25	0.50	2.07	0.040	0.080	-1.05	0.30	0.60
PANSS negative	0.53	0.60	0.60	0.65	0.52	0.52	0.12	0.91	0.91

Table S8: t-statistics and P-values for the relationship between $\overline{MS}_{global}$, $\overline{MS}_{pos}$ and $\overline{MS}_{neg}$ and the PANSS symptom scores.

We observed a positive correlation between $\overline{MS}_{pos}$ and the PANSS positive scores, which was significant before but not after FDR correction (P=0.040, P_{FDR}=0.080). The positive direction of this correlation means that patients with more positive symptoms tended to have higher MS in the regions where MS increased in patients compared to healthy control subjects. The correlation between $\overline{MS}_{neg}$ and the PANSS positive scores was in the opposite direction, in line with the fact that $\overline{MS}_{neg}$ is the mean MS in regions where MS decreased in patients compared to healthy controls, however here the correlation was not significant before or after FDR correction (P=0.30, $P_{FDR} = 0.60$). Similarly the negative correlation between $\overline{MS}_{global}$ and PANSS positive was not significant (P=0.25, $P_{FDR} = 0.50$). There were also no significant correlations between $\overline{MS}_{global}$, $\overline{MS}_{pos}$ or $\overline{MS}_{neg}$ and the PANSS negative scores.

We also had access to other measures which might be relevant, for example measures related to medication, alcohol use, cannabis use, cigarette use, education and IQ, particularly in the Maastricht and Cobre datasets (note that only information about education and IQ was available in Dublin). To explore whether any of these factors were related to $\overline{MS}_{global}$, $\overline{MS}_{pos}$ and $\overline{MS}_{neg}$ we fitted a linear model using each of these metrics in turn to predict either $\overline{MS}_{global}$, $\overline{MS}_{pos}$ or $\overline{MS}_{neg}$, with age, sex and an interaction term as co-variates. The results are given in Tables S9, S10 and S11. FDR multiple comparisons corrections were made for assessing the correlations with the various measures (8 comparisons in the Maastricht dataset, 2 comparisons in the Dublin dataset and 10 comparisons in the Cobre dataset). None of the measures were able to predict $\overline{MS}_{global}$, $\overline{MS}_{pos}$ or $\overline{MS}_{neg}$ significantly after FDR correction, apart from cannabis use in the Maastricht dataset. In Maastricht, cannabis use within the last 12 months was positively correlated with both $\overline{MS}_{global}$ and $\overline{MS}_{neg}$, with $P_{FDR} = 5 \times 10^{-4}$ and $P_{FDR} = 0.0017$, respectively. In other words, patients with higher levels of cannabis use had higher $\overline{MS}_{global}$, going against the trend for reduced $\overline{MS}_{global}$ on average across patients compared to healthy control subjects, as reported in the main text. Similarly, patients with higher levels of cannabis use tended to have higher MS in regions where MS was generally reduced in patients compared to healthy control subjects.

	$\overline{MS}_{global}$			$\overline{MS}_{pos}$			$\overline{MS}_{neg}$		
Metric	t	P	P_{FDR}	t	P	P_{FDR}	t	P	P_{FDR}
Lifetime anti-psychotic use	0.086	0.93	0.93	-1.92	0.060	0.44	0.0067	0.99	0.99
Haloperidol equivalent dose	0.93	0.36	0.71	0.48	0.64	0.65	0.18	0.86	0.99
Alcohol use	-0.24	0.82	0.93	-1.38	0.17	0.46	-0.16	0.87	0.99
Cannabis use, 12 months	**4.22**	$\mathbf{6 \times 10^{-6}}$	$\mathbf{5 \times 10^{-4}}$	0.62	0.54	0.65	**3.89**	**0.00021**	**0.0017**
Cannabis use, lifetime	2.06	0.043	0.12	1.03	0.31	0.61	1.98	0.051	0.20
Cigarette use	0.18	0.86	0.93	1.62	0.11	0.44	0.30	0.76	0.99
Education, highest level	-0.68	0.50	0.80	0.46	0.65	0.65	-0.51	0.61	0.99
Education, highest degree	-2.29	0.025	0.10	-0.75	0.46	0.65	-1.38	0.17	0.46

Table S9: t-statistics and P-values for the relationship between $\overline{MS}_{global}$, $\overline{MS}_{pos}$ and $\overline{MS}_{neg}$ and additional clinical/functional measures, in the Maastricht dataset. 'Education, highest level' refers to the highest school level reached, but can be without a certificate/diploma. 'Education, highest degree' refers to the highest level passed, with a diploma.

	$\overline{MS}_{global}$			$\overline{MS}_{pos}$			$\overline{MS}_{neg}$		
Metric	t	P	P_{FDR}	t	P	P_{FDR}	t	P	P_{FDR}
Education	1.66	0.11	0.22	-0.64	0.53	0.53	-0.38	0.71	0.76
IQ	0.38	0.71	0.71	-1.11	0.28	0.53	0.31	0.76	0.76

Table S10: t-statistics and P-values for the relationship between $\overline{MS}_{global}$, $\overline{MS}_{pos}$ and $\overline{MS}_{neg}$ and additional clinical/functional measures, in the Dublin dataset.

	$\overline{MS}_{global}$			$\overline{MS}_{pos}$			$\overline{MS}_{neg}$		
Metric	t	P	P_{FDR}	t	P	P_{FDR}	t	P	P_{FDR}
OLZ equivalent (Comed 6)	-0.43	0.67	0.74	-1.71	0.092	0.25	0.23	0.82	1.00
CPZ equivalent (Comed 7)	0.93	0.36	0.74	-1.23	0.22	0.37	-0.13	0.90	1.00
Total OLZ equivalent (Comed 8)	-0.43	0.67	0.74	-0.93	0.36	0.41	0.25	0.80	1.00
Total CPZ equivalent (Comed 9)	0.77	0.44	0.74	-1.08	0.29	0.41	-0.0013	1.00	1.00
Alcohol lifetime (scid 06)	-0.88	0.38	0.74	-1.68	0.098	0.25	-1.13	0.26	1.00
Alcohol current (scid 07)	0.49	0.63	0.74	-0.90	0.37	0.41	0.047	0.96	1.00
Cannabis lifetime (scid 10)	0.23	0.82	0.82	-2.11	0.039	0.19	-0.75	0.45	1.00
Cannabis current (scid 11)	1.48	0.15	0.70	-2.19	0.032	0.19	0.72	0.48	1.00
Highest level of education	-1.99	0.051	0.51	1.37	0.18	0.35	-0.16	0.87	1.00
WASI IQ	-1.29	0.21	0.70	0.098	0.92	0.92	0.45	0.65	1.00

Table S11: t-statistics and P-values for the relationship between $\overline{MS}_{global}$, $\overline{MS}_{pos}$ and $\overline{MS}_{neg}$ and additional clinical/functional measures, in the Cobre dataset. OLZ stands for olanzapine, CPZ stands for Chlorpromazine.

7 Yeo networks and von Economo classes

To contextualise the regional MS case-control differences, we referred them to two prior classifications of cortical areas: the von Economo atlas of cortex classified by cytoarchitectonic criteria [1]; and the Yeo atlas of cortex classified according to resting state networks derived from functional MRI [10, 5]. To do this we calculated t-statistics and corresponding P-values for regional MS differences averaged across all of the regions within a particular Yeo network or von Economo class. The results are shown in Tables S12 and S13. Across datasets there were reductions in MS in the ventral attention, frontoparietal and default mode Yeo networks, see Table S12. MS was also reduced in von Economo class 2 (association cortex).

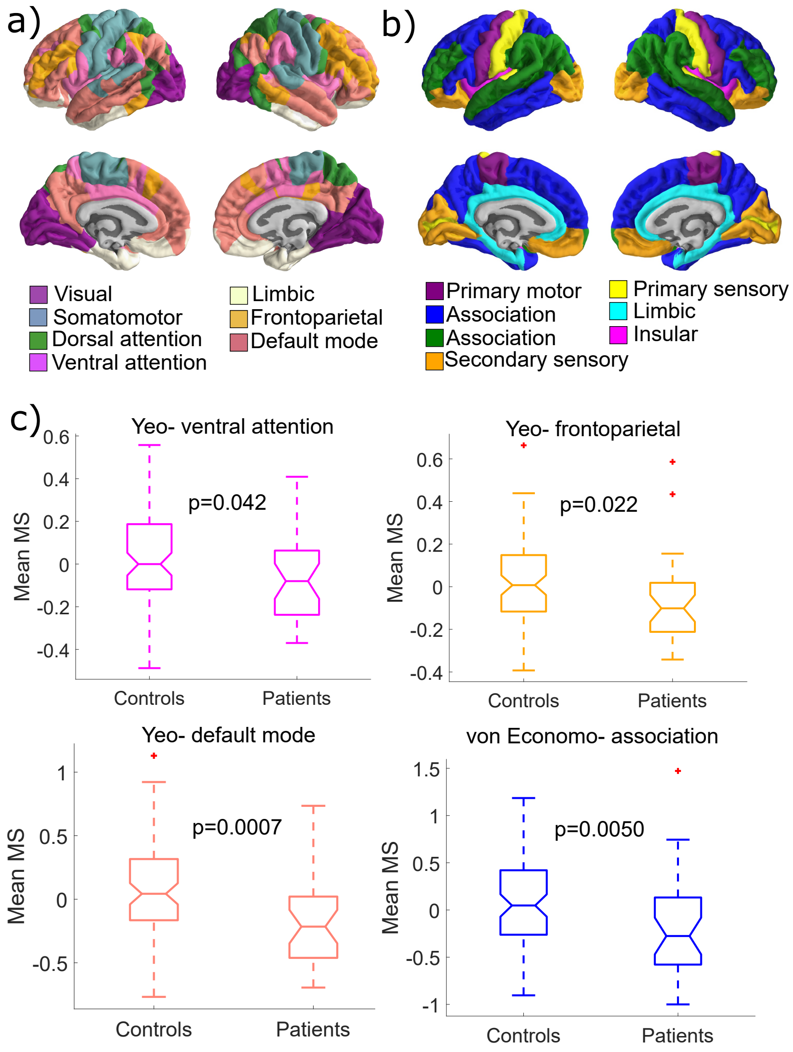

Figure S12: **Cytoarchitectonic and resting state network classification of case-control differences in regional morphometric similarity.** Brain plots coloured according to: a) the Yeo networks, b) the von Economo classes. c) Statistically significant control/patient differences in the Yeo networks and von Economo classes in the Dublin dataset. Specifically, we show the control/patient differences in Yeo network 4 (ventral attention), Yeo network 6 (frontoparietal), Yeo network 7 (default mode network) and von Economo class 2 (association cortex). The control/patient differences in these networks were also significant when results from all three datasets were combined.

Dataset	1 (visual)	2 (somatomotor)	3 (dorsal attention)	4 (ventral attention)	5 (limbic)	6 (frontoparietal)	7 (default mode network)
Maastricht t	1.1	-1.7	-1.3	-1.9	-0.20	0.89	-1.0
Maastricht p	0.26	0.090	0.20	0.058	0.84	0.38	0.30
Dublin t	2.6	0.045	1.7	-2.1	-1.7	-2.3	-3.5
Dublin p	**0.012**	0.96	0.089	**0.042**	0.094	**0.022**	**0.0007**
Cobre t	0.69	1.1	1.5	-2.0	-0.66	-3.2	-2.2
Cobre p	0.50	0.29	0.15	**0.043**	0.51	**0.0019**	**0.031**
Combined t	1.5	-0.20	0.63	-2.0	-0.85	-1.5	-2.2
Combined p (after FDR)	0.074	0.33	0.092	**0.013**	0.38	**0.0041**	**0.0038**

Table S12: t-statistics and P-values for regional MS control/patient differences averaged across each Yeo network. Results are shown for each dataset separately without FDR correction and then for the three datasets combined with FDR correction.

Dataset	1 (agranular, primary motor)	2 (association)	3 (association)	4 (secondary sensory)	5 (primary sensory)	6 (limbic)	7 (insular)
Maastricht t	-2.5	-2.5	0.43	1.7	0.45	-0.80	-0.43
Maastricht p	**0.013**	**0.012**	0.67	0.10	0.65	0.42	0.67
Dublin t	-1.1	-2.9	-1.2	2.5	2.0	-0.072	-1.7
Dublin p	0.28	**0.0050**	0.24	**0.013**	**0.050**	0.94	0.10
Cobre t	0.24	-1.2	-1.7	-0.12	1.6	-1.7	-0.21
Cobre p	0.81	0.23	0.084	0.90	0.11	0.087	0.83
Combined t	-1.1	-2.2	-0.83	1.4	1.3	-0.86	-0.77
Combined p (after FDR)	0.14	**0.0072**	0.27	0.12	0.14	0.41	0.45

Table S13: t-statistics and P-values for regional MS control/patient differences averaged across each von Economo class. Results are shown for each dataset separately without FDR correction and then for the three datasets combined with FDR correction.

8 Transcriptomic analysis

The PLS1 gene names and Z-score weights are provided in Dataset S1.

8.1 Left hemisphere t-statistics

As described in the main text, because the AHBA only includes data for the right hemisphere for two subjects, in our analyses relating gene expression to MRI data we only consider intra-hemispheric left hemisphere edges [11, 1]. We note that the t-statistics which take all (intra and inter hemispheric) edges into account are well correlated with those which take only the intra-hemispheric left hemisphere edges into account (r=0.61, $P < 0.001$ in Maastricht, r=0.71, $P < 0.001$ in Dublin and r=0.87, $P < 0.001$ in Cobre).

8.2 Spatial permutation test- correlation between PLS1 and t-statistics

As above, we perform a spatial permutation test to assess whether the correlation between PLS1 and the t-statistics from the three datasets are robust to controlling for spatial autocorrelation amongst neighboring parcels. The results are given in Table S14. The correlation between PLS1 and the t-statistics from the Dublin and Cobre datasets are robust to this permutation test.

8.3 PPI network analysis

We created PPI networks from the PLS- and PLS+ gene sets using the software STRING version 10.5 [12], with the highest confidence value of 0.9. We calculated GO enrichments for biological

Datasets	Pearson r	Pearson P-value	Spatial permutation P-value
Maastricht	0.0060	0.94	0.55
Dublin	0.49	< 0.001	< 0.001
Cobre	0.37	< 0.001	0.020

Table S14: Pearson correlation values (r) for the correlations between t-statistics from the three datasets, alongside the corresponding Pearson correlation P-value and a P-value calculated using spatial permutation.

processes and KEGG pathway enrichments of the PLS- and PLS+ genes using the software DAVID [13, 14], with a background of 15745 brain-expressed genes. The background gene list is provided in Dataset S2 and was calculated by excluding probes which did not exceed the background noise in the AHBA dataset (intensity based filtering), as described by [15]. We used code from [15], with options.probeSelections = 'maxIntensity', inline with the maximum intensity approach used to derive our regional gene expression values [16].

8.3.1 PPI network from genes with $Z < -3$

In the main text we showed GO enrichments for biological processes in the PPI network obtained from genes with $Z < -3$ (Figure 3). Two KEGG pathways are also enriched, as shown in Figure S13.

The full, high resolution PPI network from the genes with $Z < -3$, with the gene names labelled and coloured by GO enrichments for biological processes is given in Dataset S3. Significant GO enrichments for biological processes are listed in Dataset S1.

8.3.2 PPI network from genes with $Z > 3$

As reported in the main text, there were 1979 genes with $Z > 3$, 1802 of which are recognised by STRING. The resulting PPI network has 2808 edges, compared to an expected number of edges of 2542, giving a PPI enrichment P-value $< 10^{-6}$. The PPI network is shown in Figure S14, coloured by its GO enrichment for the biological process 'nucleic acid metabolic process'. The full, high resolution PPI network from the genes with $Z > 3$, with labelled gene names is given in Dataset S4.

8.3.3 PPI networks with $Z > 4$ and $Z < -4$

In order to calculate a PPI network, it was necessary to threshold the genes. The bootstrapped distribution of gene Z-scores is plotted in Figure S15. Our choice of $Z > 3$ and $Z < -3$ (highlighted on the Z-score distribution below in green) was somewhat arbitrary, although we note that if we convert the Z-scores to P-values (using a two-tailed test) and apply an FDR correction across all 20647 genes, these Z-scores correspond to $P_{FDR} < 0.014$ and $P_{FDR} < 0.025$ respectively, hence in both cases $P_{FDR} < 0.05$ ($P_{FDR} = 0.05$ is highlighted on the Z-score distribution in blue). Therefore we verified that our key results are robust to applying the more stringent threshold $|Z| < 4$ (highlighted in red), which gives much shorter gene lists, namely 807 genes with $Z > 4$ and 289 genes with $Z < -4$, corresponding to $P_{FDR} < 0.00080$ and $P_{FDR} < 0.0023$ respectively.

As before, both networks exhibited significantly more edges than expected by chance. More precisely, there were 807 genes with $Z > 4$, 740 of which were recognised by STRING. The resulting PPI network had 544 edges, compared to an expected number of edges of 484, giving a PPI enrichment P-value=0.0037. There were 289 genes with $Z < -4$, 260 of which were recognised by STRING. The resulting PPI network had 136 edges, compared to an expected number of edges of 82, giving a PPI enrichment P-value=$2.0e-8$. The PPI networks are plotted in Figures S16 and S17.

The PPI network from genes with $Z > 4$ was not significantly enriched for any KEGG pathways or GO biological processes. The PPI network from genes with $Z < -4$ was significantly enriched for three KEGG pathways- 'neuroactive ligand-receptor interaction', 'retrograde endocannabinoid signaling' and 'nicotine addiction', as well as several GO biological processes, including 'nervous system development' and 'adenylate cyclase-modulating G-protein coupled receptor signaling pathway'.

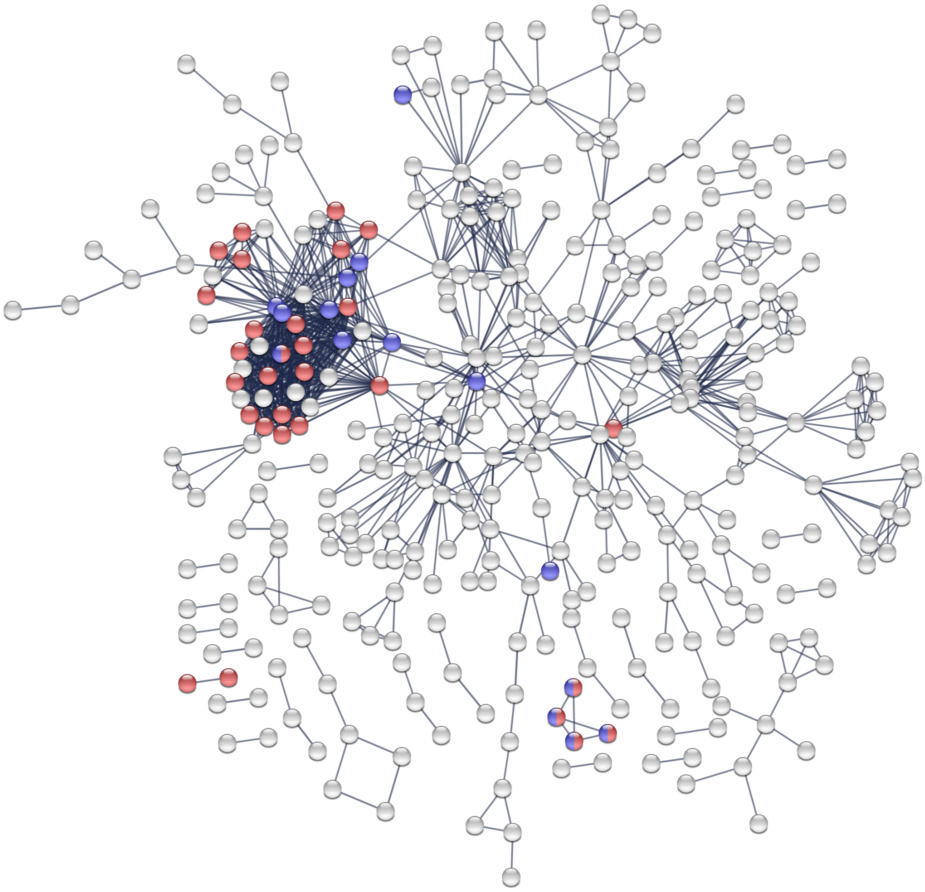

Figure S13: KEGG pathway enrichments, for PPI network from the PLS- gene set ($Z < -3$). Genes involved in the KEGG pathway ‘neuroactive ligand-receptor interaction’ are highlighted in red, genes involved in the KEGG pathway ‘retrograde endocannabinoid signaling’ are highlighted in blue.

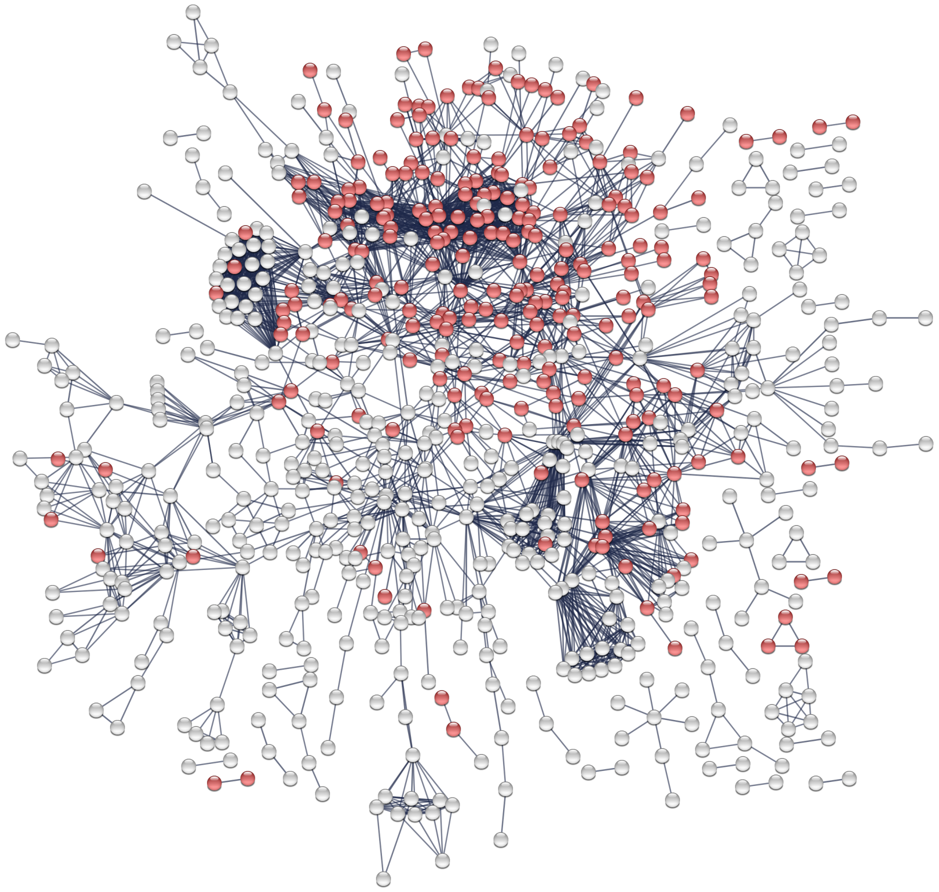

Figure S14: PPI network from PLS+ gene set ($Z > 3$). Genes highlighted in red are genes which belong to the enriched GO biological process- 'nucleic acid metabolic process'

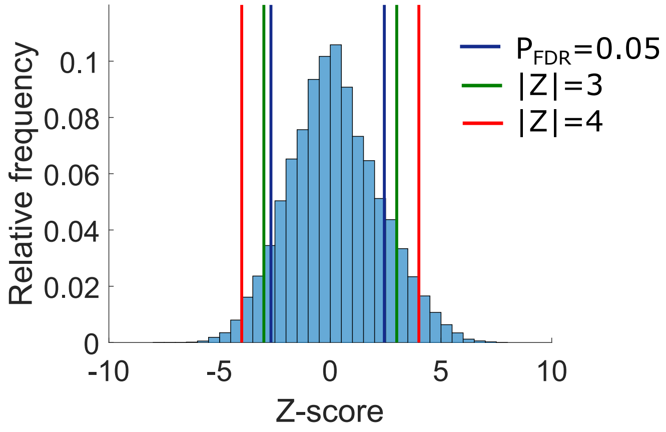

Figure S15: The bootstrapped distribution of the gene Z-scores. Lines showing $|Z| = 3$, $|Z| = 4$ and $P_{FDR} = 0.05$ are shown in green, red and blue respectively. We note that the cut-off for $|Z| < 3$ is more stringent than $P_{FDR} < 0.05$.

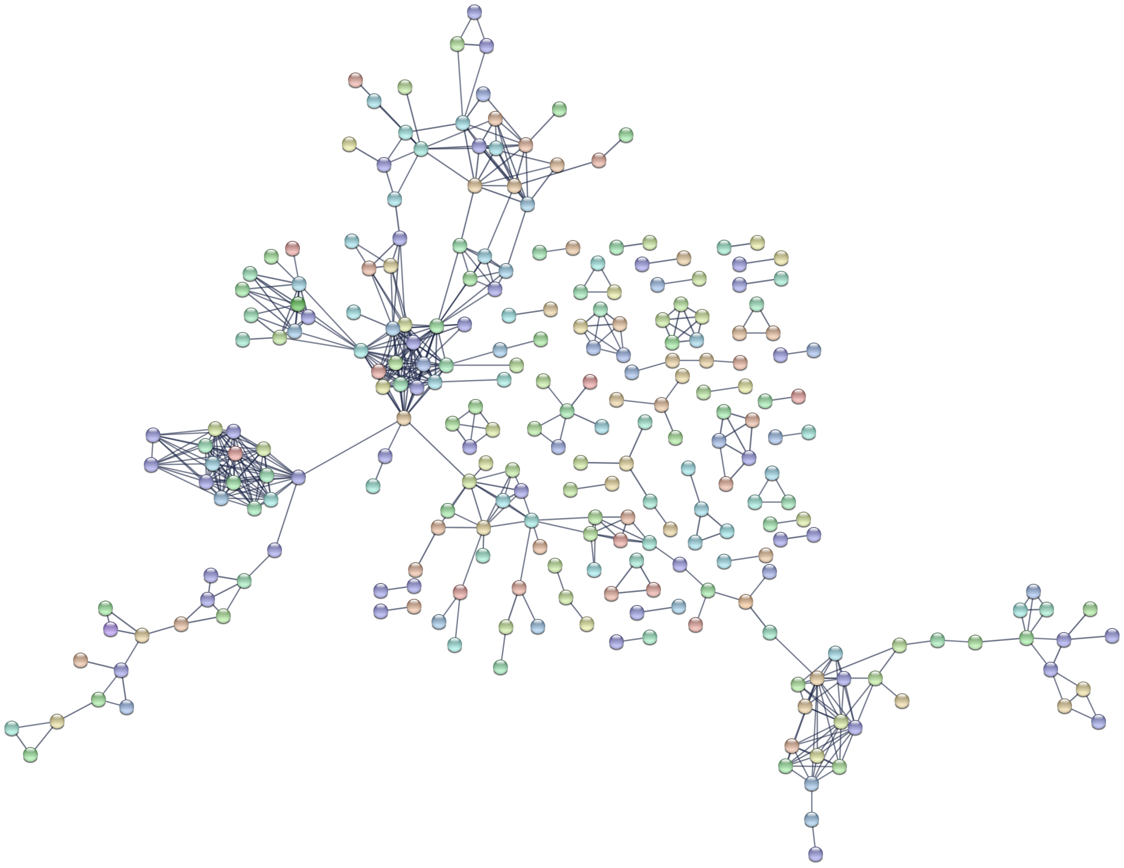

Figure S16: PPI network from genes with $Z > 4$. Colored nodes indicate query proteins and first shell interactors (as per the STRING default settings).

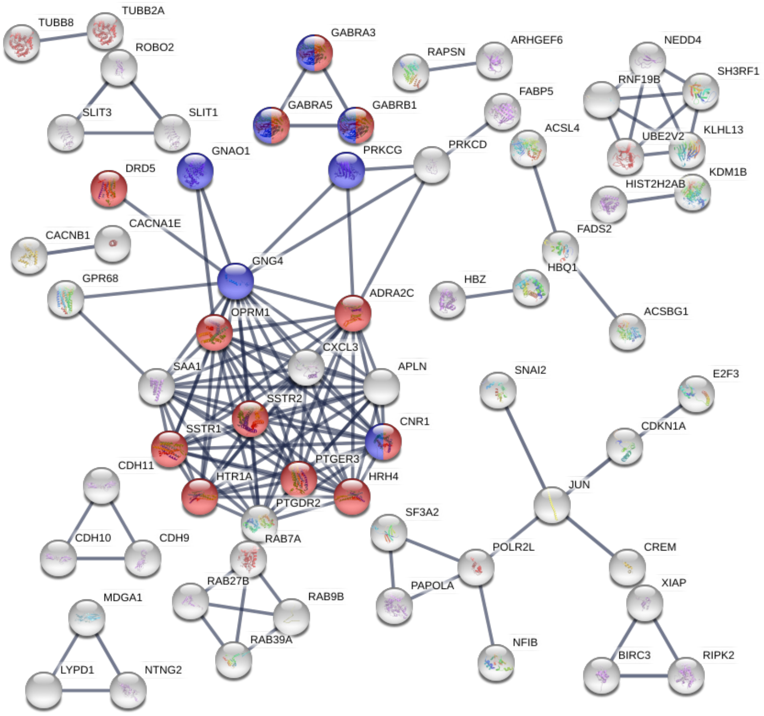

Figure S17: PPI network from genes with $Z < -4$. Genes highlighted in red and blue belong to the enriched KEGG pathways ‘neuroactive ligand-receptor interaction’ and ‘retrograde endocannabinoid signaling pathway’, respectively.

8.4 Gandal gene enrichments and specificity

In the main text we report that in PLS1, genes which are over expressed in regions of increased and decreased MS in patients are enriched for the down and up regulated Gandal genes, respectively. The P-values given in the main text for the Gandal gene enrichments are FDR corrected to account for the fact that we calculated enrichments at both the top and the bottom of each gene list (two comparisons).

Gandal et al [17] also includes lists of genes which are up and down regulated in the brain in a number of other disorders, namely bipolar disorder (BD), alcoholism (AAD), autism spectrum disorder (ASD) and major depressive disorder (MDD). Results for enrichments of these gene lists in PLS 1 are shown in Table S15. As in the main text, we use the background of brain-enriched genes. P-values are shown after FDR correction to account for the two tests made- at the top of PLS1 and at the bottom of PLS1. Our results show that the enrichment of genes which are up/down regulated in schizophrenia is not specific to abnormal gene expression in schizophrenia. We note that there is substantial overlap between the lists of genes which are up/down regulated in the different psychiatric disorders, e.g. 20% of genes up-regulated in ASD were also up-regulated in schizophrenia.

	Top of PLS1	Bottom of PLS1
AAD- up regulated	< 0.001	×
AAD- down regulated	×	×
ASD- up regulated	×	< 0.001
ASD- down regulated	< 0.001	×
BD- up regulated	×	0.0026
BD- down regulated	×	×
MDD- up regulated	×	×
MDD- down regulated	×	×
SZ- up regulated	×	< 0.001
SZ- down regulated	< 0.001	×

Table S15: P-values for the enrichment of PLS 1 for lists of genes from several brain disorders obtained from the Gandal paper. P-values > 0.05 are not shown and P-values are FDR corrected.

8.5 PsychENCODE and Fromer gene enrichments

In the main text we show that genes recently reported to be down- and up- regulated in schizophrenia [17] were enriched among the most postive and negative weighted genes on PLS1, respectively. To test the reproducibility of that result, we calculated the equivalent enrichments using two alternative large-scale transcriptional datasets: Fromer et al, 2016 [18] and the PsychENCODE study [19]. We note that there are substantial overlaps between the samples used to generate these three datasets, in particular all of the samples used in Fromer et al [18] were included in the PsychENCODE study, and the samples used in Gandal et al [17] and PsychENCODE were also substantially overlapping. In addition, there are significant overlaps between the genes included in the lists from all three studies, see Table S16, although there are also many non-overlapping genes. The enrichment results are given in Table S17. Up-regulated genes from Fromer et al [18] and PsychENCODE were both enriched in the negatively weighted PLS1 genes, reproducing the result from Gandal et al [17]. Down-regulated genes from Fromer et al [18] were enriched in the positively weighted PLS1 genes, reproducing the result from Gandal et al [17], although down-regulated genes from PsychENCODE were not (P=0.19 before FDR correction, P=0.38 after FDR correction).

Gene lists	No. overlap genes	No. genes expected to overlap	No. overlap genes/expected overlap
Gandal [17] up-regulated (849 genes) and Fromer [18] up-regulated (302 genes)	39	12	3.3
Gandal [17] down-regulated (1194 genes) and Fromer [18] down-regulated (345 genes)	76	20	3.8
Gandal [17] up-regulated (849 genes) and PsychENCODE [19] up-regulated (2450 genes)	444	101	4.4
Gandal [17] down-regulated (1194 genes) and PsychENCODE [19] down-regulated (2371 genes)	377	137	2.8
Fromer [18] up-regulated (302 genes) and PsychENCODE [19] up-regulated (2450 genes)	143	36	4.0
Fromer [18] down-regulated (345 genes) and PsychENCODE [19] down-regulated (2371 genes)	182	40	4.6

Table S16: Table with details about overlapping genes in the lists of up/down regulated schizophrenia genes. The number of genes expected to overlap is estimated as the number of genes in list 1 multiplied by the number of genes in list 2, divided by 20647 (the total number of genes in our analyses).

	Top of PLS1	Bottom of PLS1
Gandal et al 2018 up-regulated [17]	×	< 0.001
Gandal et al 2018 down-regulated [17]	< 0.001	×
Fromer et al up-regulated [18]	×	< 0.001
Fromer et al down-regulated [18]	< 0.001	×
PsychENCODE up-regulated [19]	×	< 0.001
PsychENCODE down-regulated [19]	×	×

Table S17: P-values for the enrichment of PLS1 for lists of genes from [17], [18] and [19]. P-values > 0.05 are not shown for readability and P-values are FDR corrected.

8.6 GAD

We also tested for significance of schizophrenia risk genes from the Genetic Association Database (GAD) [20, 21], which provides curated summary data on candidate gene and GWAS studies from published papers. To do this we use the same Method as for the gene lists from [17]. The GAD schizophrenia risk genes were downloaded from [21] and were significantly enriched among genes negatively weighted on PLS1 (permutation test, $P = 0.015$ after FDR correction), and not among genes positively weighted on PLS1 ($P > 0.05$).

Remarkably, a PPI network of the GAD schizophrenia genes shows a cluster of genes which is extremely similar to the cluster of genes in our PLS1 genes with $Z < -3$, as shown in Figure S18, adding confidence to the suggestion that this cluster of genes is highly relevant to schizophrenia. Figure S19 shows the GAD PPI network with the PLS1 genes with $Z < -3$ and $Z > 3$ highlighted. The genes with $Z < -3$ tend to be found in a different region of the GAD PPI network to the genes with $Z > 3$, suggesting that our imaging results might differentiate between two sets of biological processes underlying schizophrenia.

8.7 GWAS analyses

Summary statistics from a meta-analysis of GWAS results from CLOZUK and PGC data were obtained from [22]. To test PLS-derived genes for enrichment, we performed a gene set analysis on PLS+/PLS- using MAGMA v1.07b [23]. First, gene-wide P-values were calculated by combining the P-values of all SNPs inside genes, using a window of 35 kb upstream and 10 kb downstream of each gene to capture SNPs in approximate regulatory regions [24]. We then performed one-tailed competitive gene set analysis and gene property analysis, using a background list of brain-expressed genes.

For the PGC+CLOZUK genes we performed three tests using the magma software [23]: gene set analyses of the PLS+ and PLS- genes as well as a gene property analysis which compared all of

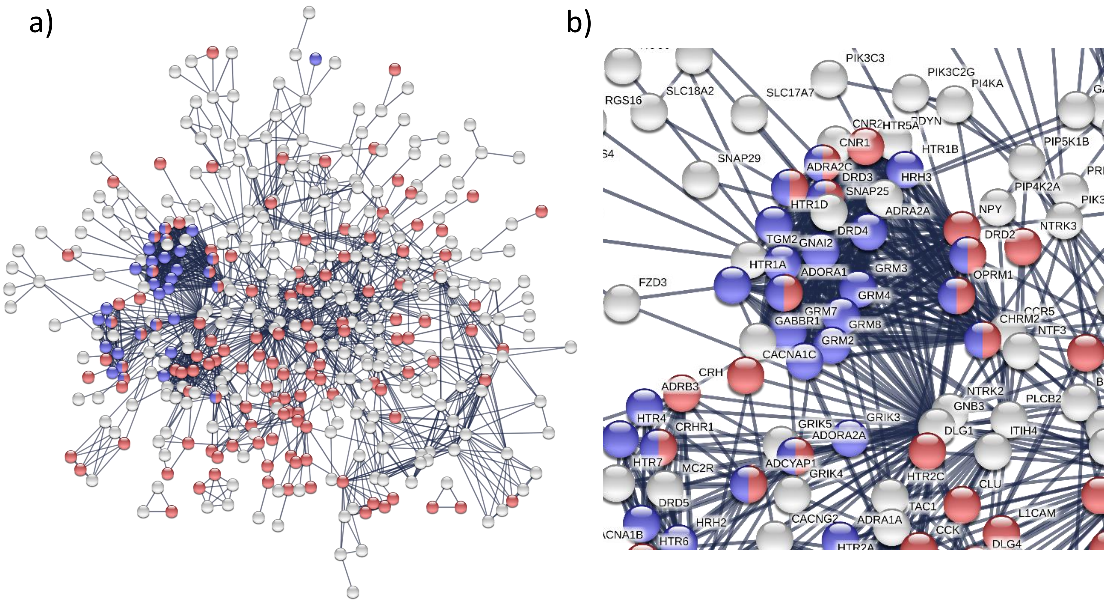

Figure S18: a) GAD PPI network, with GO enrichments for the terms 'nervous system development' and 'adenylate cyclase-modulating G-protein coupled receptor signaling pathway' highlighted in red and blue respectively. b) Zoom in on the cluster which closely resembles the cluster in the PPI network of PLS1 $Z < -3$ genes.

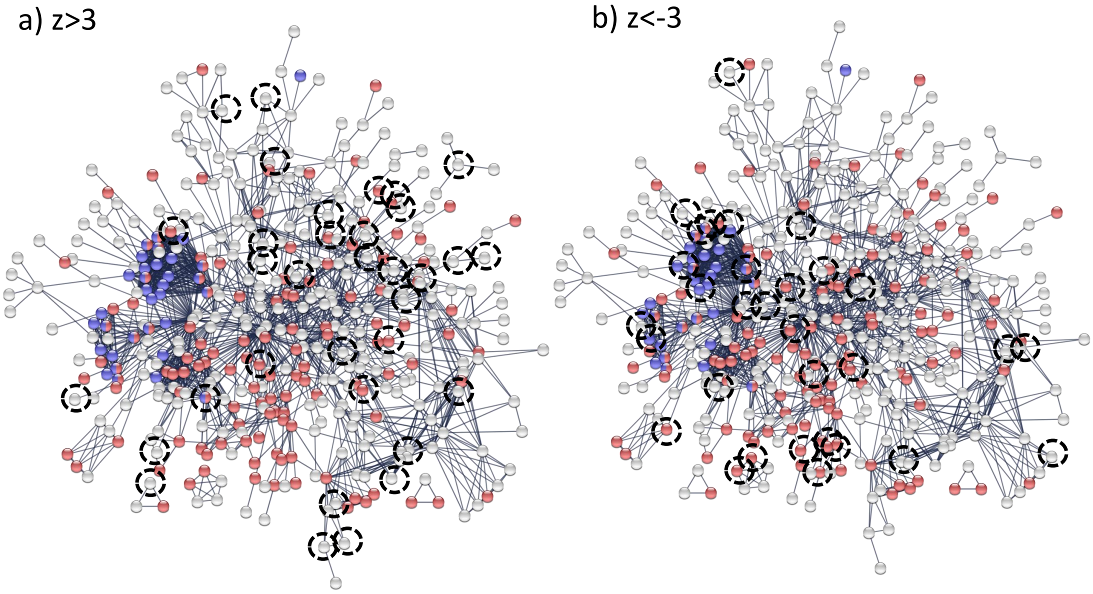

Figure S19: GAD PPI network, highlighting PLS1 genes with a) $Z > 3$ and b) $Z < -3$. GO enrichments for the terms 'nervous system development' and 'adenylate cyclase-modulating G-protein coupled receptor signaling pathway' are highlighted in red and blue respectively.

the (absolute) Z-scores from PLS1 with the P-values from the PGC+CLOZUK GWAS summary statistics. None of the P-values were significant either after or before FDR correction (before FDR correction, $P = 0.071$, $P = 0.94$, and $P = 0.93$ for gene set analysis for genes with $Z > 3$, $Z < -3$ and the gene property analysis, respectively).

8.8 Discussion of GPCR gene cluster

The PPI network from the genes which are over-expressed in regions of decreased MS is enriched for a number of relevant GO biological processes and KEGG pathways. Interestingly, the enrichments for the GO term "adenylate cyclase-modulating G-protein coupled receptor signaling pathway" and both significantly enriched KEGG pathways "neuroactive ligand-receptor interaction" and "retrograde endocannabinoid signaling" were concentrated in a tight cluster of the PPI network. Here we discuss this gene cluster in more detail. We note that the cluster includes multiple genes previously linked to schizophrenia from diverse lines of evidence across a number of genotypic and phenotypic levels. For example, polymorphisms of the *DRD5*, *OPRM1*, *CNR1*, *NOS* and *HTR7* genes have each been associated with susceptibility to schizophrenia [25, 26, 27, 28, 29], whilst polymorphism of *PDYN* was suggested to alter risk for schizophrenia via an epistatic interaction with the Gly allele of *DRD3* gene [30]. Microarray screening and real-time PCR of lymphocyte gene expression identified decreasing *NPY1R* and *GNAO1* in individuals with schizophrenia compared to unaffected family controls [31]. Complete genome sequencing in monozygotic twins discordant for schizophrenia showed differences in *GNG2* [32]. Centrally, mRNA studies in post-mortem brain tissue showed alterations in *PTGER3*, *S1PR1*, *ITPR2* and *EDNRB* [33, 17]. Several genes in this cluster have also been implicated in therapeutic approaches to schizophrenia, including *DRD4* which codes for the dopamine receptor 4 and is a target for drugs that treat schizophrenia and Parkinson's disease [34]. *HTR1A* codes for the serotonin 1A receptor (or 5-HT1A receptor) and the *5-HT1A* receptor partial agonist properties of a number of atypical antipsychotics have been shown to enhance their clinical efficacy [35]. *NTSR1* is a high-affinity receptor for neurotensin, which has been shown to selectively modulate dopaminergic neurotransmission and which was found to be reduced in the CSF and post-mortem brain tissue in schizophrenia [36, 37]. Central administration of neurotensin was also observed to produce effects similar to those of atypical antipsychotics [38]. Finally, *ADRA2C* is a candidate gene for schziophrenia because it binds clozapine, an atypical antipsychotic medication widely prescribed for treatment-resistant schizophrenia [39]. *PLCB2* was reported as a candidate causal gene in a recent genome-wide association study (GWAS) of PGC and CLOZUK samples [22]. We note that many of the other genes listed above were not identified in the most recent GWAS studies. Nevertheless, their involvement further down the causal pathway, at the epigenetic level is still mechanistically revealing and potentially useful in practice. Indeed, the remarkable density of therapeutically relevant genes in this small cluster suggest that surrounding genes may deserve further attention.

8.9 Yeo networks and von Economo classes

In Section 7 of the SI we showed that MS is significantly reduced in patients compared to control subjects in the ventral attention, frontoparietal and default mode Yeo networks (networks 4, 5 and 6), as well as in von Economo association cortex (class 2). In order to explore how our gene expression model varies in the different Yeo networks and von Economo classes, in Figure S20 we plot the PLS1 scores in each of the Yeo networks and von Economo classes, and the corresponding mean t-statistics (averaged across datasets) for comparison. One-way balanced ANOVA tests give $P < 0.001$ and $P = 0.0016$ for the variation in PLS scores across the Yeo networks and von Economo classes respectively, and $P < 0.001$ and $P < 0.001$ for the variation in mean t-statistics, suggesting that there are significant differences in both the PLS scores and the mean t-statistics across the Yeo networks and the von Economo classes. From inspection of Fig. S20, we note that the differences in PLS1 scores between the Yeo networks show a similar pattern to the differences in t-statistics between the Yeo networks. Namely, PLS1 and the mean t-statistic are both highest and positive in the Yeo visual network (network 1), whilst we observe negative values in the ventral attention, limbic, frontoparietal and default mode networks (4, 5, 6 and 7). Likewise, the differences in PLS1 scores between the von Economo classes show a similar pattern to the differences in t-statistics between the von Economo classes. Here, both PLS1 and the mean t-statistic are most positive in primary and secondary sensory cortices (von Economo classes 5 and 4), whilst they

exhibit negative values in primary motor, association, limbic and insular cortices (classes 1, 2, 6 and 7).

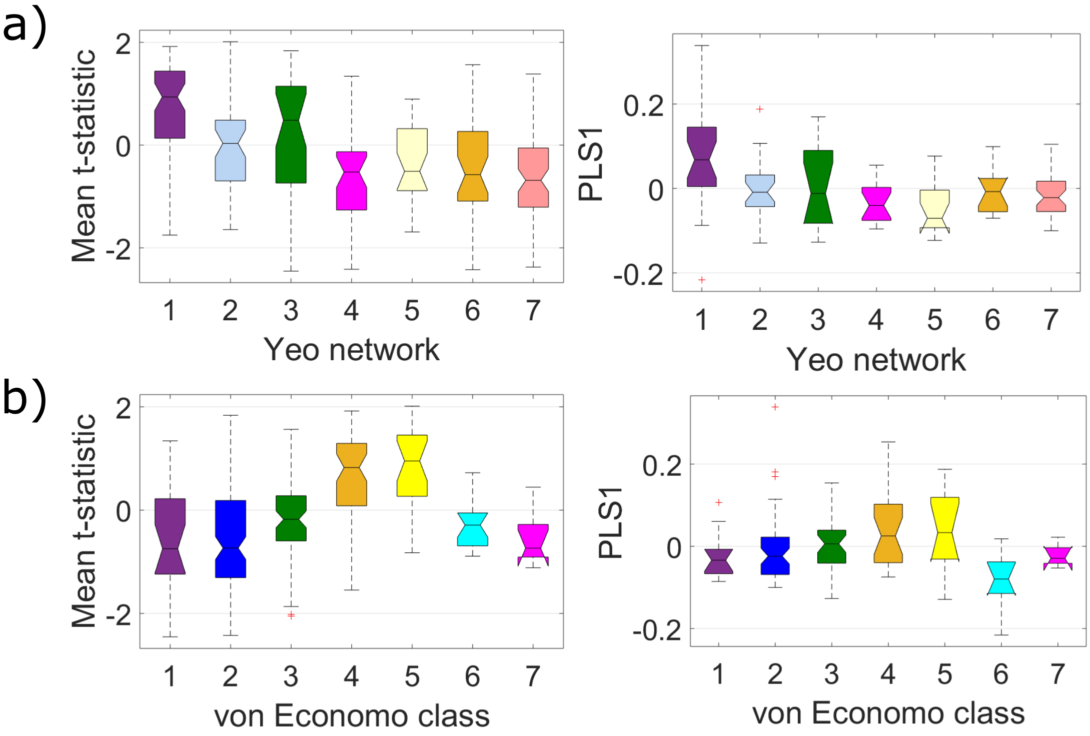

Figure S20: Box plot showing the mean t-statistics (averaged across datasets) and the corresponding PLS1 scores in each of the a) Yeo networks and b) von Economo classes.

References

[1] K. Whitaker, P. Vértes, R. Romero-Garcia, F. Vasa, M. Moutoussis, G. Prabhu, N. Weiskopf, M. Callaghan, K. Wagstyl, T. Rittman, R. Tait, C. Ooi, J. Suckling, B. Inkster, P. Fonagy, R. Dolan, P. Jones, I. Goodyer, NSPN, and E. Bullmore, "Adolescence is associated with genomically patterned consolidation of the hubs of the human brain connectome," *PNAS*, vol. 113, pp. 9105–9110, 2016.

[2] K. Whitaker, "UCHANGE structural neuroimaging pipeline." dx.doi.org/10.17504/protocols.io.eu5bey6, 2017.

[3] P. Habets, M. Marcelis, E. Gronenschild, M. Drukker, and J van Os for GROUP, "Reduced cortical thickness as an outcome of differential sensitivity to environmental risks in schizophrenia," *Biological Psychiatry*, vol. 69, pp. 487–494, 2011.

[4] A. Rosen, D. Roalf, K. Ruparel, J. Blake, K. Seelaus, L. Villa, R. Ciric, and et al, "Quantitative assessment of structural image quality," *NeuroImage*, vol. 169, pp. 407–418, 2017.

[5] F. Váša, J. Seidlitz, R. Romero-Garcia, K. J. Whitaker, G. Rosenthal, P. Vértes, M. Shinn, A. Alexander-Bloch, P. Fonagy, R. Dolan, P. Jones, I. Goodyer, N. Consortium, O. Sporns, and E. T. Bullmore, "Adolescent tuning of association cortex in human structural brain networks," *Cerebral Cortex*, vol. 28, pp. 281–294, 2018.

[6] D. Lakens, "Calculating and reporting effect sizes to facilitate cumulative science: a practical primer for t-tests and ANOVAs," *Front Psychol.*, vol. 4, p. 863, 2013.

[7] J. Cohen, *Statistical Power Analysis for the Behavioral Sciences*. Routledge Academic, 1988.

[8] T. van Erp, A. Preda, D. Ngyuen, L. Faziola, J. Turner, J. Bustillo, A. Belger, K. Lim, S. McEwan, J. Voyvodic, D. Mathalon, J. Ford, S. Potkin, and FBIRN, "Converting positive and negative symptom scores between PANSS and SAPS/SANS," *Schizophrenia Research*, vol. 152, pp. 289–294, 2014.

[9] S. Leucht, J. Kane, W. Kissling, J. Hamann, E. Etschel, and R. Engel, "What does the PANSS mean?," *Schizophrenia Research*, vol. 79, pp. 231–238, 2005.

[10] B. Yeo, F. Krienen, J. Sepulcre, M. Sabuncu, D. Lashkari, M. Hollinshead, J. Roffman, J. Smoller, L. Zöllei, J. Polimeni, B. Fischl, H. Liu, and R. Buckner, "The organization of the human cerebral cortex estimated by intrinsic functional connectivity," *J. Neurophysiol.*, vol. 106, pp. 1125–1165, 2011.

[11] P. Vértes, T. Rittman, K. Whitaker, R. Romero-Garcia, F. Váša, M. Kitzbichler, K. Wagstyl, P. Fonagy, R. Dolan, P. Jones, I. Goodyer, the NSPN Consortium, and E. Bullmore, "Gene transcription profiles associated with inter-modular hubs and connection distance in human functional magnetic resonance imaging networks," *Philosophical Transactions of the Royal Society B*, vol. 371, p. 1705, 2016.

[12] D. Szklarczyk, J. Morris, H. Cook, M. Kuhn, S. Wyder, M. Simonovic, A. Santos, N. Doncheva, A. Roth, P. Bork, L. Jensen, and C. von Mering, "The STRING database in 2017: quality-controlled protein-protein association networks, made broadly accessible.," *Nucleic Acids Res.*, vol. 45, pp. 362–368, 2017.

[13] D. Huang, B. Sherman, and R. Lempicki, "Systematic and integrative analysis of large gene lists using david bioinformatics resources," *Nature Protoc.*, vol. 4, pp. 44–57, 2009.

[14] D. Huang, B. Sherman, and R. Lempicki, "Bioinformatics enrichment tools: paths toward the comprehensive functional analysis of large gene lists," *Nucleic Acids Res.*, vol. 37, pp. 1–13, 2009.

[15] A. Arnatkevičiūte, B. Fulcher, and A. Fornito, "A practical guide to linking brain-wide gene expression and neuroimaging data," *NeuroImage*, vol. 189, pp. 353–367, 2018.

[16] R. Romero-Garcia, K. Whitaker, F. Váša, J. Seidlitz, M. Shinn, P. Fonagy, R. Dolan, P. Jones, I. Goodyer, N. Consortium, E. Bullmore, and P. Vértes, "Structural covariance networks are coupled to expression of genes enriched in supragranular layers of the human cortex," *NeuroImage*, vol. 171, pp. 256–267, 2018.

[17] M. Gandal, J. Haney, V. Parikshak, Neelroopand Leppa, G. Ramaswami, C. Hartl, A. Schork, V. Appadurai, A. Buil, T. Werge, C. Liu, K. White, C. M. Consortium, P. Consortium, iPSYCH BROAD Working Group, S. Horvath, and D. Geschwind, "Shared molecular neuropathology across major psychiatric disorders parallels polygenic overlap," *Science*, vol. 359, pp. 693–697, 2018.

[18] M. Fromer, P. Roussos, S. Sieberts, J. Johnson, D. Kavanagh, T. Perumal, D. Ruderfer, and et al, "Gene expression elucidates functional impact of polygenic risk for schizophrenia," *Nature Neuroscience*, vol. 19, pp. 1442–1453, 2016.

[19] M. Gandal, P. Zhang, E. Hadjimichael, R. Walker, C. Chen, S. Liu, H. Won, H. van Bake, M. Varghese, Y. Wang, A. Shieh, J. Haney, S. Parhami, J. Belmont, M. Kim, P. Moran Losada, Z. Khan, J. Mleczko, Y. Xia, R. Dai, D. Wang, Y. Yang, M. Xu, K. Fish, P. Hof, J. Warrell, D. Fitzgerald, K. White, A. Jaffe, P. Consortium, M. Peters, M. Gerstein, C. Liu, L. Iakoucheva, D. Pinto, and D. Geschwind, "Transcriptome-wide isoform-level dysregulation in ASD, schizophrenia, and bipolar disorder," *Science*, vol. 362, p. 6420, 2018.

[20] K. Becker, K. Barnes, T. Bright, and A. Wang, "The genetic association database," *Nature Genetics*, vol. 36, pp. 431–432, 2004.

[21] A. Rouillard, G. Gundersen, N. Fernandez, Z. Wang, C. Monteiro, M. McDermott, and A. Ma'ayan, "The harmonizome: a collection of processed datasets gathered to serve and mine knowledge about genes and proteins.," *Database*, vol. 2016, p. baw100, 2016.

[22] A. Pardiñas, P. Holmans, A. Pocklington, V. Escott-Price, S. Ripke, N. Carrera, S. Legge, S. Bishop, D. Cameron, M. Hamshere, J. Han, L. Hubbard, A. Lynham, K. Mantripragada, E. Rees, J. MacCabe, S. McCarroll, B. Baune, G. Breen, E. Byrne, U. Dannlowski, T. Eley, C. Hayward, N. Martin, A. McIntosh, R. Plomin, D. Porteous, N. Wray, A. Caballero, D. Geschwind, L. Huckins, D. Ruderfer, E. Santiago, P. Sklar, E. Stahl, H. W. Won, E. Agerbo, T. Als, O. Andreassen, M. Bækvad-Hansen, P. Mortensen, C. Bøcker Pedersen, J. Børglum, Andersand Bybjerg-Grauholm, S. Djurovic, N. Durmishi, M. Giørtz Pedersen, V. Golimbet, J. Grove, D. Hougaard, M. Mattheisen, E. Molden, O. Mors, M. Nordentoft, M. Pejovic-Milovancevic, E. Sigurdsson, T. Silagadze, C. Søholm Hansen, K. Stefansson, H. Stefansson, S. Steinberg, S. Tosato, T. Werge, G. Consortium, C. Consortium, D. Collier, D. Rujescu, G. Kirov, M. Owen, M. O'Donovan, and J. Walters, "Common schizophrenia alleles are enriched in mutation-intolerant genes and in regions under strong background selection," *Nat. Genet.*, vol. 50, pp. 381–389, 2018.

[23] C. de Leeuw, J. Mooij, T. Heskes, and D. Posthuma, "MAGMA: Generalized gene-set analysis of GWAS data," *PLoS Comput Biol*, vol. 11, p. e1004219, 2015.

[24] The Network and Pathway Analysis Subgroup of the Psychiatric Genomics Consortium, "Psychiatric genome-wide association study analyses implicate neuronal, immune and histone pathways," *Nat. Neurosci.*, vol. 18, pp. 199–209, 2015.

[25] Y. Zhao, M. Ding, H. Pang, X. Xu, and B. Wang, "Relationship between genetic polymorphisms in the DRD5 gene and paranoid schizophrenia in northern Han Chinese," *Genetics and Molecular Research*, vol. 13, pp. 1609–1618, 2014.

[26] E. Gouvêa, S. Filho, V. Ota, V. Mrad, A. Gadelha, R. Bressan, Q. Cordeiro, and S. Belangero, "The role of the CNR1 gene in schizophrenia: a systematic review including unpublished data," *Braz J Psychiatr*, vol. 39, pp. 160–171, 2017.

[27] O. Serý, R. Prikryl, L. Castulík, and F. St'astný, "A118G polymorphism of OPRM1 gene is associated with schizophrenia," *J Mol Neurosci.*, vol. 41, pp. 219–222, 2010.

[28] H. Weber, D. Klamer, F. Freudenberg, S. Kittel-Schneider, O. Rivero, C. Scholz, J. Volkert, J. Kopf, J. Heupel, S. Herterich, R. Adolfsson, A. Alttoa, A. Post, H. Grußendorf, A. Kramer, A. Gessner, B. Schmidt, S. Hempel, C. Jacob, J. Sanjuán, M. Moltó, K. Lesch, M. Freitag, L. Kent, and A. Reif, "The genetic contribution of the NO system at the glutamatergic post-synapse to schizophrenia: Further evidence and meta-analysis," *European Neuropsychopharmacology*, vol. 24, pp. 65–85, 2014.

[29] M. Ikeda, N. Iwata, T. Kitajima, T. Suzuki, Y. Yamanouchi, Y. Kinoshita, and N. Ozaki, "Positive association of the serotonin 5-HT7 receptor gene with schizophrenia in a japanese population," *Neuropsychopharmacology*, vol. 31, pp. 866–871, 2006.

[30] M. Ventriglia, L. Bocchio Chiavetto, C. Bonvicini, G. Tura, S. Bignotti, G. Racagni, and M. Gennarelli, "Allelic variation in the human prodynorphin gene promoter and schizophrenia," *Neuropsychobiology*, vol. 46, pp. 17–21, 2002.

[31] M. Vawter, E. Ferran, B. Galke, K. Cooper, W. Bunney, and W. Byerley, "Microarray screening of lymphocyte gene expression differences in a multiplex schizophrenia pedigree," *Schizophrenia Research*, vol. 67, pp. 41–52, 2004.

[32] C. Castellani, M. Melka, J. Gui, A. Gallo, R. O'Reilly, and S. Singh, "Post-zygotic genomic changes in glutamate and dopamine pathway genes may explain discordance of monozygotic twins for schizophrenia," *Clinical and Translational Medicine*, vol. 6, p. 43, 2017.

[33] B. Tang, C. Capitao, B. Dean, and E. Thomas, "Differential age- and disease-related effects on the expression of genes related to the arachidonic acid signaling pathway in schizophrenia," *Psychiatry Res.*, vol. 196, p. 201–206, 2012.

[34] L. Yet, "Five-membered Rings with Two Heteroatoms, each with their Fused Carbocyclic Derivatives," *Comprehensive Heterocyclic Chemistry III*, vol. 4, pp. 1–141, 2008.

[35] H. Rollema, Y. Lu, A. Schmidt, J. Sprouse, and S. Zorn, "5-HT1A receptor activation contributes to ziprasidone-induced dopamine release in the rat prefrontal cortex," *Biological Psychiatry*, vol. 48, pp. 229–237, 2000.

[36] J. Austin, P. Buckland, A. Cardno, N. Williams, B. Spurlock, G Hoogendoorn, S. Zammit, G. Jones, R. Sanders, L. Jones, G. McCarthy, S. Jones, N. Bray, P. McGuffin, M. Owen, and M. O'Donovan, "The high affinity neurotensin receptor gene (NTSR1): comparative sequencing and association studies in schizophrenia," *Molecular Psychiatry*, vol. 5, pp. 552–557, 2000.

[37] R. Sharma, P. Janicak, G. Bissette, and C. Nemeroff, "CSF neurotensin concentrations and antipsychotic treatment in schizophrenia and schizoaffective disorder," *Am J Psychiatry*, vol. 154, pp. 1019–1021, 1997.

[38] C. Nemeroff, B. Levant, B. Myers, and G. Bissette, "Neurotensin, antipsychotic drugs and schizophrenia," *Ann NY Acad Sci*, vol. 668, pp. 146–156, 1992.

[39] J. Feng, J. Zheng, J. Gelernter, H. Kranzler, E. Cook, D. Goldman, I. Jones, N. Craddock, L. Heston, L. Delisi, L. Peltonen, W. Bennett, and S. Sommer, "An in-frame deletion in the alpha(2C) adrenergic receptor is common in African–Americans.," *Mol. Psychiatry*, vol. 6, pp. 168–72, 2001.
